# Specific Amino Acid Residues in the Three Loops of Snake Cytotoxins Determine Their Membrane Activity and Provide a Rationale for a New Classification of These Toxins

**DOI:** 10.3390/toxins16060262

**Published:** 2024-06-04

**Authors:** Peter V. Dubovskii, Yuri N. Utkin

**Affiliations:** Shemyakin-Ovchinnikov Institute of Bioorganic Chemistry, Russian Academy of Sciences, 16/10 Miklukho-Maklaya Str., 117997 Moscow, Russia; utkin@ibch.ru

**Keywords:** three-finger toxins, cobra cytotoxins/cardiotoxins, structure–function relationship, conformational equilibrium, cis/trans isomerism of peptide bonds, NMR spectroscopy, membrane activity, synergism, phospholipase A2

## Abstract

Cytotoxins (CTs) are three-finger membrane-active toxins present mainly in cobra venom. Our analysis of the available CT amino acid sequences, literature data on their membrane activity, and conformational equilibria in aqueous solution and detergent micelles allowed us to identify specific amino acid residues which interfere with CT incorporation into membranes. They include Pro9, Ser28, and Asn/Asp45 within the N-terminal, central, and C-terminal loops, respectively. There is a hierarchy in the effect of these residues on membrane activity: Pro9 > Ser28 > Asn/Asp45. Taking into account all the possible combinations of special residues, we propose to divide CTs into eight groups. Group 1 includes toxins containing all of the above residues. Their representatives demonstrated the lowest membrane activity. Group 8 combines CTs that lack these residues. For the toxins from this group, the greatest membrane activity was observed. We predict that when solely membrane activity determines the cytotoxic effects, the activity of CTs from a group with a higher number should exceed that of CTs from a group with a lower number. This classification is supported by the available data on the cytotoxicity and membranotropic properties of CTs. We hypothesize that the special amino acid residues within the loops of the CT molecule may indicate their involvement in the interaction with non-lipid targets.

## 1. Introduction

Animal venoms originated and evolved as a means of hunting and/or defense against predators. They are complex mixtures of proteins, peptides, and other organic and inorganic compounds. The components of animal venoms usually called toxins have a high selectivity of action on certain body systems and high efficiency of interactions with their biological targets. The most widely known venomous animals are snakes, scorpions, and spiders, whose venoms contain mainly toxins of a polypeptide nature. Animal toxins vary greatly in their molecular mass, spatial structure, and biological activity. They may have enzymatic activity (proteinases, phospholipase A2, etc.) or lack it. Among the variety of non-enzymatic toxins present in the venoms of various organisms, toxins with a three-finger structure (TFT) play a prominent role [1,2,3,4,5]. Among TFTs, there are toxins, e.g., neurotoxins (NTs) of narrowly targeted action that interact with protein receptors [5,6,7,8,9]. Other representatives of TFTs, cytotoxins (CT), or cardiotoxins interact with a wider range of biomolecules, which include primarily lipids as well as, at least, carbohydrates and proteins [9,10,11,12]. In this paper, the structure–function relationships in CTs will be considered. First, the conformational equilibria of CTs in model membrane systems and their membrane-active properties will be outlined. This will allow us to determine the residues and their combinations influencing the membrane activity of CTs. Finally, the extent to which membrane activity determines the cytotoxic activity of CTs will be discussed.

## 2. CT Structure and Biological Effects

**Figure 1:**
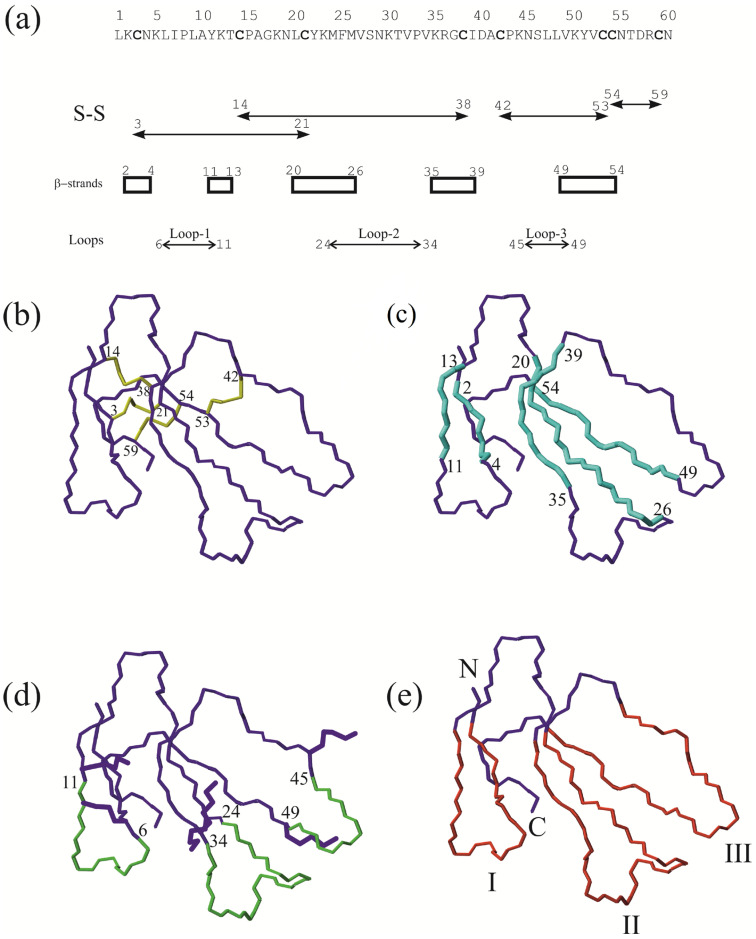
Structural organization of CTs and terminology used in this paper on the example of one of the latest CT structures (cytotoxin 13 from *Naja naja*, pdb code 7QHI) obtained by X-ray diffraction. (**a**) Amino acid sequence of the toxin. The numbering of amino acid residues is indicated above. Cys residues are marked bold. Below the amino acid sequence, the disulfide bonds, the beta-strand boundaries, and those of the loops of the molecule are indicated. Spatial models of the toxin with highlighted (**b**) disulfide bonds (yellow), (**c**) beta-strands (cyan), (**d**) loops (green), and (**e**) fingers (red). In addition to the loops, the side chains of lysine residues (5 and 12, 23 and 35, 44 and 50, and encircling the loop-1, 2, and 3, respectively) are shown in the panel (**d**). The orientation of the model is the same in all the panels. The N- and C-termini are shown in panel (**e**) only. Here, the finger numbers are indicated below in Roman numerals.

CT are beta-structured polypeptides with five beta strands extending from the head of the molecule stabilized by four disulfide bonds, as shown in Figure 1a,b. The termini of the beta strands (Figure 1a,c) are connected by fragments of amino acid sequences that belong to a category of the secondary structure called loops (Figure 1a,d) [13,14]. Beta strands in combination with loops form fingers (Figure 1e). Their total number gives the name to the whole structural family. CT structures have been well characterized by both X-ray and NMR spectroscopy. The role of individual amino acid residues in maintaining the spatial structure and its stabilization has been revealed [15,16,17,18,19,20,21,22,23]. The PDB data bank currently contains 36 CT structures, including those with and without ligands, obtained under different conditions. The first structure dates back to 1990 [24] and it represents the refinement of a model obtained in 1987 [25]. The most recent structure was published in 2022 [26]. The noted discrepancies between the X-ray and NMR structures of CTs [2] have been resolved by applying higher-field NMR spectrometers. The recent NMR structures take into consideration the long-lived tightly bound water molecule in the second loop [27,28], and are determined using heteronuclear ^13^C, ^15^N-chemical shifts obtained either at the natural content of these isotopes [28] or at recombinantly produced isotope-labeled CTs [29,30]. The X-ray diffraction structure of one CT (Figure 1a) (PDB code 7QHI) [26] is almost identical to the NMR structure of this toxin (6RC7) [31].

CTs are present mainly in the venoms of cobras [32,33,34,35]. Their content varies considerably. For example, the proportion of CTs in *N. nivea* venom is about 75% [10,36], while in *N. samarensis*, it does not exceed 17% [37]. The weighted average value obtained by analyzing the venom of 17 different cobra species is equal to 47% [38]. CTs are believed to target non-protein components of biological membranes [39,40,41]. An alternative viewpoint ascribing them capability to interact with some proteins, affecting their function, has not yet been supported experimentally [42,43,44]. The diversity of action targets makes toxins in the CT group extremely diverse in their effects on living cells, organs, and tissues [39]. This makes them similar to other membrane-active cytolytic peptides, such as the toxins from spiders [45], bees [46], and other insect [47,48,49,50] venom. All cytolytic peptides are linear (without disulfide bonds) polycationic peptides that form an alpha-helix when interacting with lipid membranes [51,52,53]. It is established that both CTs and latarcins destabilize the lipid bilayers of biological membranes [53]. Once incorporated into these membranes, toxins affect their mechanical properties via the modulation of the bending elastic modulus [54,55]. Thus, these peptides influence the oligomerization of the intramembrane domains of integral proteins and ion channels [56,57].

## 3. Place of CTs in the TFT Family

**Figure 2:**
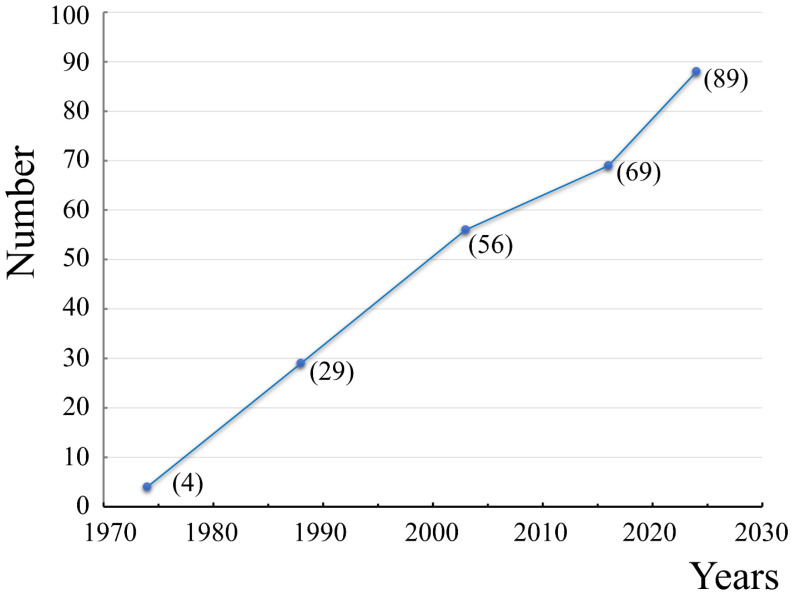
Time dependence of the number of identified CTs. The dots represent the number of CTs (indicated in parentheses), as cited in reviews and papers: Condrea, 1974 [59]; Dufton and Hider, 1988 [60]; Fry et al., 2003 [1]; Goraj et al., 2016 [61], and this paper (point with abscissa 2024). The data presented do not contain Cardiotoxin-like Basic Polypeptides (CLBP), repeats, and variants missing in the current databases.

One of the first CTs was isolated from the venom of *N. atra* and its amino acid sequence was determined in 1970 [58]. In one of the first reviews on CTs, dated by 1974, the amino acid sequences of only four CTs were given [59]. In the review by Dufton and Hider in 1988, about 50 sequences were collected (more than half of them are absent in modern databases because they were refined afterwards resulting in 1–2 amino acid substitutions) [60]. These authors divided the toxins into true CTs and their homologues. Since that time, the number of identified CTs has been increasing from year to year (Figure 2).

To date, the known CTs are listed in Table 1. More detailed information about CTs, including their physicochemical characteristics, available spatial structures in the PDB bank, alternative names and identity of CTs with different accession numbers are summarized in Table S1. Most of the CTs consist of 60 amino acid residues. Only four CTs are characterized by the His4 insertion, extending them by one amino acid residue. The former were proposed to be classified as Type IA (85 items) and the latter as Type IB (4 items) (Table 1) [1]. We have assigned to Type IB a CT named hemachatoxin with the accession code B3EWH9 [62]. All Type IB CTs are isolated from the venom of *Hemachatus haemachatus* (Ringhals cobra).

**Table 1:** The list of CTs with known amino acid sequences ^1^.

Type IA	Q02454, P60307, AAB35381.1 ^2^, Q98965, CAA90962.1 ^2^, AAB25732.1 ^2^, Q9PS33, P01447, P86382, P24780, P01445, P01446, P0CH80, P83345, P60306, Q98961, P07525, P01442, P24779, P01440, P01451, P60304, Q98959, Q98958, Q98962, Q9PST3, P79810, Q91135, O73856, Q98960, Q98957, Q91136, Q91124, O93473, Q9DGH9, O93471, Q98956, P01458, P01452, P01461, P01457, Q9PS34, P01459, P80245, P01460, P01455, P01441, P01463, P01456, P01464, P49123, P01462, P60309, P60308, P01465, P25517, P01468, P01469, P01466, Q9W6W6, P01467, P01470, P01448, P01454, P01453, P01443, Q9W6W9, O73858, O73859, P60311, E2ITZ7, P86541, P86540, A0A0U5AL91, A0A8C6XG05, P86538, A0A0U4W6K7, A0A8C6XQ33, A0A8C6XFH6, A0A8C6XFM5, A0A0U5ARS4, A0A0U5AR60, A0A0U4W6H0, P0DSN1, O93472
Type IB	P01471, P24776, P24777, B3EWH9

^1^ CT accession numbers in UniProt knowledgebase are listed; ^2^ GenBank accession numbers are indicated for these CTs.

Among several TFT groups, the closest homologs of CTs are Cardiotoxin-Like Basic Polypeptides (CLBPs). However, we treat CLBP as a separate group, just as Dufton and Hider did [60]. Both CTs and CLBPs are capable of being incorporated into lipid membranes by the three loops [63]. However, CLBPs do not perturb the lipid bilayer so strongly as CTs, and consequently manifest a lower cytotoxicity compared to CTs [64,65,66]. The aligned amino acid sequences of CLBPs are shown in Figure S1. Their phyco-chemical characteristics and database codes are given in Table S2. The boundary between CTs and CLBPs is still not completely defined. However, it has been observed that CLBPs, unlike CTs, have the ability to specifically interact with some proteins, e.g., integrins [67].

According to recent studies, the emergence of new CTs is ongoing [68]. Their recombinant alternatives, which may differ from the native CTs by the presence of additional residues at the N-terminus of the molecule [29], continue to appear, too. Judging by the trend in Figure 2, the number of identified CTs will exceed 100 toxins by 2030. This will be facilitated by the emergence of new improved techniques for analyzing snake venoms [69], as well as the analysis of their genomes [35,70,71,72]. It should be noted that in databases (e.g., UniProt knowledgebase), some CTs are deposited under other names (see Table S1, “Name” column). The term cytotoxin is also used in the names of other TFTs, in particular, beta-cardiotoxins [73]. However, beta-cardiotoxins are drastically different from CTs due to their in vivo effects. Moreover, these toxins do not show hemolytic activity characteristic to CTs. Also, within their loops, more hydrophilic residues are present compared to CTs (see, e.g., Figure 1 in [73]) and some residues invariant in CTs (e.g., Tyr22 and Pro33) are absent. Therefore, these toxins will be not considered further.

What proportion of the total number of TFTs do CTs constitute? To date, we estimate that about 900 TFTs have been collected in the UniProt knowledgebase (this excludes variants with an amino acid sequence length of less than 50 amino acid residues and identical toxins deposited under different names). CTs and CLBPs account for 103 representatives (according to Tables S1 and S2). Thus, about 11% of all TFTs are CTs/CLBPs.

**Figure 3:**
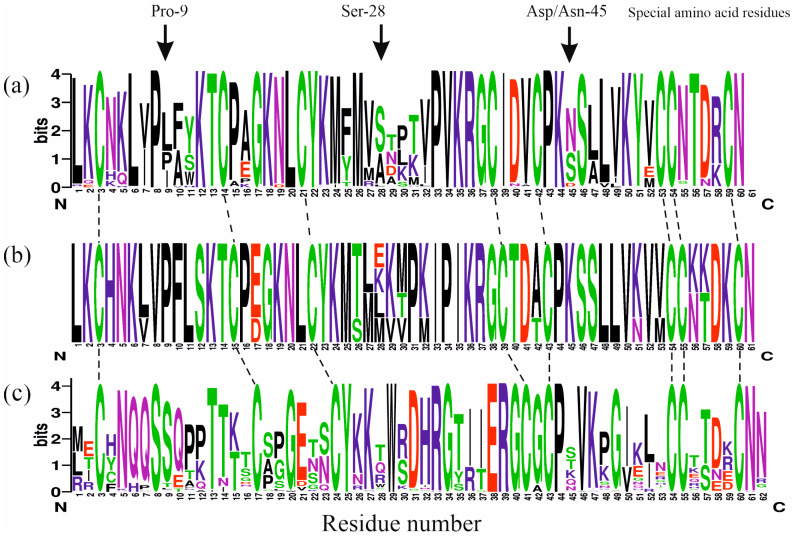
Peculiarities of the amino acid composition of CTs and short neurotoxins. Logo-sequence of IA-type CTs (**a**) and frequency plot of IB-type CTs (**b**), as well as logo-sequence of short neurotoxins (**c**) are shown. The amino acid sequences of short neurotoxins used for logo-sequencing are summarized in Table S3. The size of the letters on the vertical Y-axis (bits, left) corresponds to the conservativity of the residue. Cysteine residues that are conserved for all presented TFTs are connected by dashed lines. In panel (**a**), specific amino acid residues are indicated at the top. Logo sequences and frequency plot were obtained using the web server: https://weblogo.berkeley.edu/logo.cgi (accessed on 30 April 2024).

CTs differ from other TFTs at the level of the amino acid sequence and spatial structure [74]. In both cases, the loops are mainly involved [75]. The peculiarities of the amino acid sequence of CTs are reflected in their logo-sequence (Figure 3a,b). For comparison, the logo-sequence of short neurotoxins (NTs) is presented in this figure (Figure 3c). The amino acid sequences of the NTs used for its construction are given in Table S3. Traditionally, NTs and CTs are considered side by side. This is due to the close history of their discovery, investigation of their mechanism of action, and subsequent comparative studies [59,76,77,78,79,80,81,82]. In addition, cobra venom usually contains both representatives of these TFTs. However, the LD50 of the intravenous administration of CTs is approximately two orders of magnitude higher than for NTs [83]. Thus, the toxicity of the latter is significantly higher than that of CTs. Consequently, the ratio of NTs to CTs in the venom determines its lethality [84,85,86].

In CTs, the loops are limited by lysine residues 5 and 12, 23 and 35, 44 and 50 for the first, second, and third loops, respectively (Figure 3a). Furthermore, the CT loops contain more hydrophobic amino acid residues (Leu, Val, Met, Phe, Tyr, and Trp) than the NT loops (Figure 3c). In total, 18 residues including all the Cys residues as well as Pro8,33,43, Lys12,35,44, Leu20, Tyr22, and Arg36 are strictly conserved for CTs (Figure 3a,b). For NTs (Figure 3c), there are 16 such residues including all Cys residues as well as Ser8, Tyr25, Trp29, Gly34,40, Glu38, Arg39, and Pro44. The higher variability in the amino acid sequences is observed in the loops of NTs compared to CTs (Figure 3a–c). The combination of hydrophobic loops with flanking lysine residues is a definitive feature of CTs and other cytolysins [87]. This assumes that there are a number of requirements for the amino acid sequences of CTs. Therefore, CTs have a high degree of amino acid sequence similarity (Figure 3a,b), unlike NTs (Figure 3c). This is also because the mechanism of evolution of CTs is different from that of other TFTs. Most TFTs evolved rapidly, whereas CTs remain constrained by a negative selection [88]. This is evident from the conservatism of CT amino acid sequences, which differ in only one or two amino acid residues from each other. Considering that the common ancestor of CTs is NTs [89], CTs feature conserved residue characteristic of these TFTs [75,90]. These include Ser28, Asp29, and Asn45, Ser46, Val49 in loop-2 and -3, respectively (Figure 3a–c). The minimum percentage of homology in the identity matrix for the entire set of CTs from Table 1 is 56.7%. For the set of NTs (Table S3), the respective value is equal to 51.67%.

## 4. Conformational Equilibria of CTs in Membrane Environment and the Role of Special Amino Acid Residues

The study of the interaction of CTs with model lipid membranes has shown that the perturbation of the lipid bilayer depends on the amount of toxin bound to the membrane [91]. At low degrees of binding, the toxin is in a monomeric state, affecting mainly the viscoelastic properties of the membrane [54,92]. When the toxin content in the membrane increases, oligomerization occurs [93,94,95], probably depending on the content of anionic lipids in the membrane [96]. At the higher toxin loads, bilayer disruption occurs with the formation of lipid particles [97,98,99]. We propose to focus on the monomer stage. This will help us to formulate the definition of the special amino acid residues in CTs. For this purpose, we will consider the interaction of different CTs with detergent micelles, which are a common membrane-modeling medium [100,101,102,103]. In dodecylphosphocholine micelles, it is possible to find conditions where approximately one toxin molecule is bound to the micelle [104,105].

**Figure 4:**
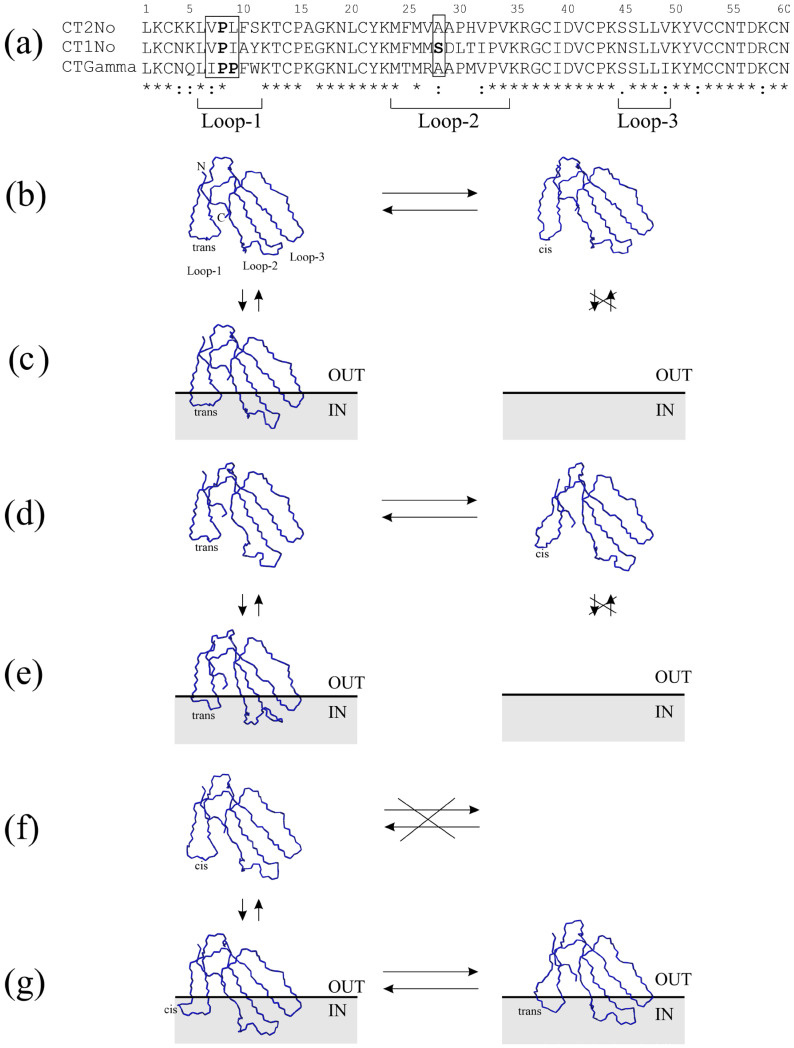
Conformational equilibrium in aqueous solution and lipid environment for CTs with one and two Pro residues in the loop-1, according to NMR data. Alignment of the amino acid sequences of the considered CTs was done using the CLUSTALO program (**a**). CT names are given on the left. Residue numbering is given at the top. The boundaries of the loops are shown at the bottom. Residue identity, similarity in hydrophilicity/hydrophobicity, or size/shape are indicated at the bottom by asterisks, colons, and dots, respectively. Pro residues in the loop-1 and Ser28 residues in the loop-2 are shown in bold. Structural changes for selected CTs characterized by differences in the residues enclosed by rectangles in the panel (**a**) are shown for CT2No (**b**,**c**), CT1No (**d**,**e**), and CTGamma (**f**,**g**). Panels show models calculated from NMR data (**b**,**c**,**e**,**f**) or predicted theoretically using Alphaphold (**f**). Conventionally, the membrane is shown as a gray rectangle with a thick line indicating membrane/water interface. The intramembrane region is labeled “IN” and the aqueous phase is labeled “OUT”. Horizontal bidirectional arrows indicate the slow conformational equilibrium between the two forms observed by NMR. Crossed horizontal arrows (panel **f**) indicate a shift in the equilibrium toward one form. Vertical bidirectional arrows correspond to the transition of the toxin from the aqueous environment to the membrane and vice versa. Crossed-out vertical arrows indicate no binding of a given form to the membrane. Models in all panels are oriented in the same way. N- and C-termini and loops are labeled only in the panel (**b**). The configuration of Val7-Pro8 (in panels **b**–**e**) or Pro8–Pro9 (panels **f**,**g**) bonds is labeled as cis/trans. PDB codes of the models shown in panel (**b**): 1CB9 (**left**), 1CCQ (**right**), in (**c**): 1FFJ (**left**), in (**d**): 1RL5 (**left**), 5LUE (**right**, this model is derived for recombinant CTs with an additional Met residue at the N-terminus; this residue has been removed), in (**e**): 5NQ4 (**left**), in (**f**): model predicted with Alphaphold (**left**), in (**g**): 1TGX (X-ray model A, **left**), the model obtained from 2CCX one via template modeling (**right**). Only the polypeptide backbones without side chains are shown.

Previously, for CT2No (Figure 4a), it was shown for the first time by NMR that in an aqueous solution, there was a slow (on the NMR time scale) exchange between two structural forms, called the major (Figure 4b, left) and minor (Figure 4b, right) forms [27].

In earlier works using ^1^H-NMR data, we were able to structurally characterize both forms and showed that they differed in the isomerism of the Val7-Pro8 peptide bond (Figure 4a; hereinafter, residue numbering is given for 60-residue-long CTs) [27]. In the major conformer (Figure 4b, left), this bond is in the trans form, while in the minor conformer (Figure 4b, right), it is in the cis form. The proportion of the latter state is about 20%. Later, for another CT, CT1No (Figure 4a, more precisely, its recombinant ^13^C,^15^N-isotope-enriched form, which differs from the native CT by the presence of an additional Met residue at the N-terminus), possessing a single Pro residue in loop-1, similar results were also obtained using three-dimensional NMR techniques (Figure 4d) [30]. Interestingly, using native CTs and ^1^H-NMR, it was shown that for both CT2No (Figure 4c, left) and CT1No (Figure 4e, left), only the major forms had the ability to incorporate into micelles of dodecylphosphocholine (DPC) [105,106]. In the minor form, the amide proton of the residue preceding Pro8 becomes 100% exposed to the aqueous phase which makes this form energetically unfavorable for interaction with the lipid membrane for both CT2No (Figure 4b, right) and CT1No (Figure 4d, right) [107]. A comparison of the structures of these forms in aqueous solution (Figure 4b, left) and micelle (Figure 4c, left) for CT2No shows that the incorporation of the toxin into the micelle is accompanied by small structural changes in the loops [105]. These changes reflect the adaptation of the loops to the membrane environment. Later, using Molecular Dynamics (MD) methods, it was possible to show that these findings extend to lipid bilayer membranes [108].

The NMR study in an aqueous solution of CTs with two Pro residues in loop-1, in particular, toxin gamma (CTGamma) from *N. pallida* (Figure 4a), showed that only one form was present in the aqueous solution (Figure 4f, left) [104]. In addition, in this form, the bond between the Pro8 and Pro9 residues is in the cis-configuration. This was established by NMR based on the chemical shift analysis for another CT with two prolines in the first loop, CTA6 from *N. atra* (Table 2) [21].

**Table 2:** CT mentioned in this and the following sections ^1^.

Amino Acid Sequence	Name	Snake	Abbreviation	Database Identifier	Group ^2^	ID No in Table S1
LKCHKLVPPVWKTCPEGKNLCYKMFMVSTSTVPVKRGCIDVCPKNSALVKYVCCSTDKCN	Cytotoxin 1	*N. annulifera*	CT1Nan	P01455	1	6
LKCHKLVPPFWKTCPEGKNLCYKMYMVATPMLPVKRGCIDVCPKDSALVKYMCCNTDKCN	Cytotoxin 2	*N. annulifera*	CT2Nan	P01462	3	10
LKCNKLIPIASKTCPAGKNLCYKMFMMSDLTIPVKRGCIDVCPKNSLLVKYVCCNTDRCN	Cytotoxin 1	*N. atra*	CT1Na	P60304	5	36
LKCNKLVPLFYKTCPAGKNLCYKMFMVSNLTVPVKRGCIDVCPKNSALVKYVCCNTDRCN	Cytotoxin 2	*N. atra*	CT2Na	P01442	5	34
RKCNKLVPLFYKTCPAGKNLCYKMFMVSNLTVPVKRGCIDVCPKNSALVKYVCCNTDRCN	Cytotoxin 4	*N. atra*	CT4Na	P01443	5	45
LKCNQLIPPFYKTCAAGKNLCYKMFMVAAQRFPVKRGCIDVCPKSSLLVKYVCCNTDRCNN	Cytotoxin 6	*N. atra*	CT6Na	Q98965	4	13
LKCNQLIPPFYKTCAAGKNLCYKMFMVAAPKVPVKRGCIDVCPKSSLLVKYVCCNTDRCN	Cytotoxin A6	*N. atra*	CTA6	P80245	4	14
LKCNKLIPLAYKTCPAGKNLCYKMFMVSNKTVPVKRGCIDVCPKNSLLVKYVCCNTDRCN	Cytotoxin 2	*N. kaouthia*	CT2Nk	P01445	5	29
LKCNKLIPLAYKTCPAGKNLCYKMFMVSNKTVPVKRGCIDACPKNSLLVKYVCCNTDRCN	Cytotoxin 3	*N. kaouthia*	CT3Nk	P01446	5	30
LKCNQLIPPFWKTCPKGKNLCYKMTMRAAPMVPVKRGCIDVCPKSSLLIKYMCCNTNKCN	Cytotoxin 1	*N. mossambica*	CT1Nm	P01467	4	19
LKCNQLIPPFWKTCPKGKNLCYKMTMRGASKVPVKRGCIDVCPKSSLLIKYMCCNTDKCN	Cytotoxin 2	*N. mossambica*	CT2Nm	P01469	4	17
LKCNRLIPPFWKTCPEGKNLCYKMTMRLAPKVPVKRGCIDVCPKSSLLIKYMCCNTNKCN	Cytotoxin 3	*N. mossambica*	CT3Nm	P01470	4	20
LKCNKLIPIAYKTCPEGKNLCYKMMLASKKMVPVKRGCINVCPKNSALVKYVCCSTDRCN	Cytotoxin 4	*N. mossambica*	CT4Nm	P01452	5	42
LKCKKLIPLFSKTCPEGKNLCYKMTMRLAPKVPVKRGCIDVCPKSSFLVKYECCDTDRCN	Cytotoxin 5	*N. mossambica*	CT5Nm	P25517	8	83
LKCNKLVPLFYKTCPKGKNLCYKMYMVAAPTVPVKRGCINVCPKNSLVLKYECCNTNKCN	Cytotoxin	*N. naja*	newCT	-	7	69
LKCNKLIPLAYKTCPAGKNLCYKMYMVSNKTVPVKRGCIDVCPKNSLVLKYECCNTDRCN	Cytotoxin 1	*N. naja*	CT1Nn	P01447	5	26
LKCNKLVPLFYKTCPAGKNLCYKMYMVATPKVPVKRGCIDVCPKSSLVLKYVCCNTDRCN	Cytotoxin 2	*N. naja*	CT2Nn	P01440	8	73
LKCNKLIPLAYKTCPAGKDLCYKMYMVSNKTVPVKRGCIDVCPKNSLLVKYECCNTDRCN	Cytotoxin 7	*N. naja*	CT7Nn	P86382	5	27
LKCNKLVPLAYKTCPAGKNLCYKMYMVANKKVPVKRGCIDVCPKKSLLVKYECCNTDRCN	Sagitoxin	*N. sagittifera*	CTSNs	P83345	8	72
LKCNKLVPLFYKTCPAGKNLCYKMYMVATPKVPVKRGCIDVCPKSSLLVKYVCCNTDRCN	Cytotoxin 2b	*N. sputatrix*	CT2bNsp	O73856	8	75

^1^ The full list of CTs is given in Table S1; ^2^ see Table 3 for details. The situation with loop-2 is somewhat different for CT1No. In this toxin, the shape of loop-2 changes markedly during the transition from the aqueous phase (Figure 4d, left) to the micelle (Figure 4e, left) [106]. The Ser28-Asp29 residues (Figure 4a, enclosed in a box) appear displaced toward the membrane/water interface (Figure 4e, left). It has not been possible to observe such a change in the shape of loop-2 in X-ray structures of CTs having a Ser28 residue in loop-2 until recently. The selection of crystallization conditions for one of the CTs (Figure 1) allowed us to reproduce this structural feature of CTs [109]. Using MD, we were able to show that this structural change is characteristic of all CTs with a Ser28 residue upon their incorporation into the lipid membrane [26]. However, the change in the shape of loop-2 does not affect its ability to coordinate a long-lived water molecule [106]. The latter is characteristic of this loop for all CTs in an aqueous solution [28,30].

Although no structure calculation was performed based on NMR data, an X-ray structure was obtained for this toxin (Figure 4g, left). An interesting feature of this form is the banana-twisted shape of loop-1. Probably, this loop shape is important to avoid cis-bond burial when the toxin is incorporated into the membrane. However, an early NMR model of a similar toxin (Figure 4g, right) does not support the presence of such a bend [110]. The CTGamma structure predicted using Alphaphold [111] also lacks such a bend (Figure 4f, left). Perhaps subsequent structural studies of such a CT will clarify this issue. However, the fact remains undeniable that due to the additional Pro residue in this loop, the equilibrium is shifted toward the “minor” form with a cis-configuration of the bond (Figure 4f, left). Of course, calling this state “minor” is not quite correct. It was minor for CT2No (Figure 4b, right) and CT1No (Figure 4d, right). 

Upon embedding CTGamma in the DPC micelle, two slow-exchanging forms were observed (Figure 4g) [104]. This was an obstacle for the calculation of the spatial structure of these forms, especially under the conditions where the NMR signals are broadened, which is characteristic for the micellar medium. However, considering the data obtained for other CTs (Figure 4b–e), it becomes obvious that this exchange is caused by the energetic disadvantage of the cis-form in the membrane. The only variant of the conformational equilibrium is the transition of the cis form of the Pro8-Pro9 bond in the loop-1 to its trans configuration (Figure 4g). As we have seen, such a transition for Val7-Pro8 bonds in CT2No and CT1No is a slow process in aqueous solution (Figure 4b,d). 

Thus, consideration of the above conformational equilibria leads to the conclusion that at least in loop-1 of CTs, there is a residue (or a combination of the residues, e.g., Pro8–Pro9) that makes the incorporation of CT into the lipid membrane energetically unfavorable. So far, the presence of a similar residue has been identified for CTs in loop-2. This residue is Ser28 (Figure 4a). The identification of the role of Ser28 in the interaction of CTs with lipid membranes led to the division of all CTs into the P- and S-type [112]. CTs lacking Ser28 were classified as P-type. As it turned out later, the special residues (Asp/Asn) are also present in loop-3 of CT molecules (see next section). We refer to all these residues as special amino acid residues (Figure 3a,b) because they affect the incorporation of CTs into the lipid membrane. Their identification allows us to predict the membrane activity of CTs and to propose their new classification.

## 5. Classification of CTs, According to Their Membrane Activity

**Figure 5:**
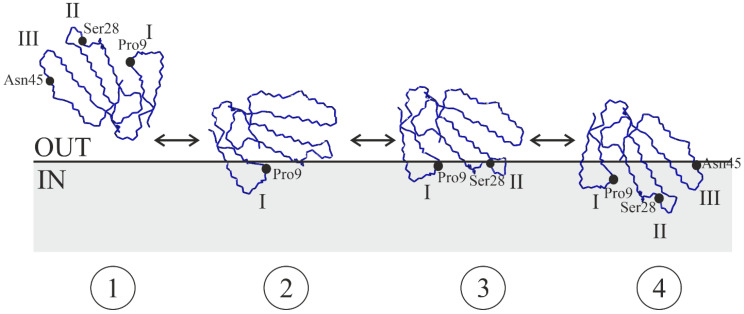
Model of the interaction of CTs with lipid bilayer and the role of special amino acid residues. There are 4 stages for embedding of the CT molecule in the lipid bilayer (schematically depicted as a gray rectangle, the thick line indicates the membrane/water interface). They are numbered with Arabic numerals in circles under each state. At stage 1, the molecule is in the aqueous phase. Stages 2–4 correspond to the incorporation of loop-1 only, loop-1 and -2, and all three loops, respectively. For all stages, only the polypeptide backbone is shown. Roman numerals number the fingers of the toxin molecule. The orientation of the fingers at stage 1 is chosen arbitrarily. When transitioning to stage 2, the toxin is oriented in a specific way for loop-1 to be incorporated into the membrane. The bidirectional arrows between the positions of the toxin on the membrane depend on the presence of the special amino acid residues in the loops (see text for the details). Cα-atoms of special amino acid residues are indicated by a black circle.

In addition to understanding the role of specific amino acid residues in the loops of CTs in their interaction with lipid membranes, it is necessary to consider the role of the loops themselves. The hierarchy between loops in the cytolytic activity of CTs was first highlighted by the cytolytic assay developed by Ma et al. [113]. They constructed a series of chimeric toxin molecules by swapping the loops between neurotoxin and cardiotoxin molecules from *Naja sputatrix*. The comparison of the cytolytic activities of the recombinant chimeric toxins demonstrated that the first two loops made the major contribution to its lytic activity. The hierarchy between the loops in the incorporation of CTs into a lipid membrane is consistent with the previous observation of the hydrophobicity gradient between the loops [40]. Specifically, the hydrophobicity decreases from loop-1 to loop-2 and then to loop-3. Indeed, according to a recent MD study [108] of the incorporation of the CT2No molecule into the POPC membrane, loop-1 is the first to be incorporated. This is followed by the incorporation of loop-2. In the last step, loop-3 is partitioned. Taking into account this hierarchy, we obtain a model of the interaction of CTs with the lipid membrane (Figure 5). 

Considering the conformational equilibria described in the previous section and illustrated in Figure 4, we come to the following conclusions regarding the role of specific amino acid residues in the loops. Namely, the presence of Pro9 in loop-1 results in a cis-bond between the Pro8–Pro9 residues at stage 1. This is unfavorable for the interaction with the membrane because it requires a rearrangement of the conformation of this bond to a trans configuration. Therefore, the equilibrium is shifted towards stage 1, i.e., the aqueous phase, compared to the toxins in which the Pro-9 residue is absent.

If the Ser28 residue is present in loop-2, such a toxin, when embedded in the membrane by loop-1, has difficulty in transitioning from stage 2 to stage 3 because loop-2 requires adaptation to the membrane environment. Thus, compared to a toxin with the Pro30 residue instead of Ser28, loop-2 of which does not require a conformational rearrangement, the equilibrium is shifted towards stage 2 in the toxin with Ser28. This is consistent with the earlier suggestion that S-type toxins interact with membranes predominantly through loop-1 [112]. However, more recent studies of the membrane insertion of such toxins using MD in a coarse-grained approximation allow us to conclude that stages 3 and 4 are also populated [106]. 

Are special amino acid residues present in loop-3? Earlier studies indicated that the main contribution to the lytic activity of CTs belongs to loops-1 and -2 (e.g., [113]). However, by studying the interaction with the liposomes of as many as seven CTs, we were able to determine the role of loop-3 in this activity. Our set of CTs included CT1Nn, CT7Nn, CT2Nk, CT2Nn, CT1Nan, CT2Nan, and one newly characterized CT, newCT (Table 2) [68].

Among the seven CTs, two (CT1Nan and CT2Nan) were characterized by the presence of a pair of Pro residues in the first loop. One of them (CT1Nan) contained a Ser residue in loop-2 and the other (CT2Nan), a Pro residue. Of the remaining five CTs, three (CT1Nn, CT7Nn, and CT2Nk, similar to CT1Nan) were of the S-type and two (CT2Nn and newCT, similar to CT2Nan) were of the P-type. The latter two CTs differed in the composition of loop-3: one (CT2Nn) contained a pair of Ser45-Ser46 residues and the other (newCT) was characterized by an Asn45-Ser46 pair in this loop. All of the above CTs caused the leakage of fluorescent dye from liposomes composed of DOPC/DOPG (1:1). Its content was strictly individual for each CT and depended on the combination of specific amino acid residues in each of the three loops of the CT. The two CTs with a Pro pair in loop-1 were the least active, and of this pair, the toxin with a Pro residue in loop-2 (CT2Nan) was more active than with a Ser residue in this loop (CT1Nan). The activity increased for CTs with one Pro residue in loop-1. Moreover, loop-2 began to play a role here: all S-type CTs (CT1Nn, CT7Nn, and CT2Nk) were less active than the P-type CTs (CT2Nn and newCT). Of the latter two, the activity was higher for CTs with a pair of Ser-Ser residues in loop-3 (CT2Nn) than for CTs with a pair of Asn-Ser (newCT). Thus, the alternation of activities for the selected CTs confirms the conclusions drawn from the study of the conformational equilibria of CTs in micelles (Figure 4 and Figure 5). Indeed, the membrane activity of CTs depends on the presence of specific amino acid residues not only in loops 1 and 2, but also in loop-3, in particular at position 45 (Figure 2 and Figure 5).

Based on the data presented above, depending on the presence of special residues in certain loops, eight combinations of special amino acid residues can be obtained, that is, all CTs can be divided into eight groups (Table 3). In the group with the lowest number, all special residues are present. In the group with the maximal number, they are absent. The membrane activity increases along with increasing the group number.

**Table 3:** Classification of CTs according to the presence of the special amino acids within their loops ^1^.

Special Amino Acid ^2^/Loop Number	CTs’ Groups (Abbreviation ^3^)							
	1	2	3	4	5	6	7	8
Pro9/Loop-1	**P**	**P**	**P**	**P**	**O**	**O**	**O**	**O**
Ser28/Loop-2	**S**	**S**	**O**	**O**	**S**	**S**	**O**	**O**
Asn/Asp45/Loop-3	**X**	**O**	**X**	**O**	**X**	**O**	**X**	**O**

^1^ Valid for a broad spectrum of lipid membranes, including mammalian, bacterial plasma membranes, and artificial phospholipid compositions. ^2^ Note that numbering of amino acid residues is given for 60-residue-long CTs, constituting their majority; only five CTs are 61-residue long and the numbering of critical amino acid residues is given for 60-residue-long members in Figure 3. ^3^ The abbreviation of the name of each group consists of the three letters in the corresponding column, e.g., group-1 becomes PSX, etc., O designates Omission, X stands for Asn/Asp.

The distribution of all of the known CTs among the proposed groups is presented in Table 4 (its extended version, containing amino acid sequences of the toxins, is presented in the Supplementary Materials, Table S4).

**Table 4:** Distribution of all known CTs among the proposed groups.

Group Number	Identification Codes of CTs	Number of the Members in the Groups
1	P01458, P01461, P01457, P01459, P01460, P01455, P01456	7
2	This group is empty	0
3	P01463, P01464, P01462, P01465, P01466	5
4	P80245, P49123, P01468, Q9W6W6, P01467, P01470, P0DSN1, Q98965, P01469	9
5	AAB35381.1, CAA90962.1, AAB25732.1, Q9PS33, P01447, P86382, P24780, P01445, P01446, P0CH80, P60306, Q98961, P01442, P01451, P60304, Q98958, P79810, Q91135, Q98957, Q91136, P01452, P01454, P01453, P01443, O73858, O73859, P60311, P86540, A0A0U5AL91, A0A8C6XG05, P86538, A0A0U4W6K7, A0A8C6XQ33, A0A0U5ARS4, A0A0U5AR60, A0A0U4W6H0	36
6	P07525, Q98956, P01448, Q9W6W9, P83345, A0A8C6XFH6, A0A8C6XFM5	7
7	P24779, Q98959, Q98962, Q98960, Q9DGH9, newCT	6
8	Q02454, P60307, P01440, Q9PST3, O73856, Q91124, O93473, O93471, Q9PS34, P01441, P60309, P60308, P25517, E2ITZ7, P01471, B3EWH9, P24777, P24776, P86541, O93472	20
		In total: 90 items

It should be noted that group 2 is empty. We do not exclude that members of this group may appear later, as new CTs are identified.

The proposed classification of CTs extends their division into either P- or S-types, made by Wu WG et al. about 30 years ago [112]. Since then, due to an absence of alternatives, this division has been used by researchers while comparing cytotoxic and other activities of CTs [61,114,115,116,117,118,119]. Wu WG et al. investigated the membrane activities of nine CTs (CT1Ns, CT1Na, CT2Na, CT3Na, CT4Na, CT1Nm, CT2Nm, CT4Nm, and CT5Nm) (Table 2) and one CLBP A5 (Table S2). They used sonicated vesicles formed from zwitterionic sphingomyelin. However, it is known that CTs interact poorly with zwitterionic phospholipids compared to their anionic counterparts [120,121,122]. This may be the reason that only two groups of CTs were identified. CTs with Ser28 residue were categorized as the S-type. The absence of a Ser residue at this position correlates with the presence of a Pro30 residue, which is not specific in our classification. All CTs with Pro30 were classified as the P-type. In our set of CTs (Table 1), there are only three exceptions to this rule: these are CTs lacking both a Ser28 residue and Pro30 (CT6Na, CTSNs, and CT2Nm, Table 2). We have shown that the division into S- and P-types is independent of the lipid composition [92]. Dufton and Hider previously divided CTs into four subgroups, A, B, C, and D, depending on the presence/absence of Pro residues at positions 9 and 30 (numbering for 60-membered CTs) [60]. Taking into account that Pro9 is a special residue in our classification and that Pro30 correlates with the absence of Ser28, their classification into groups was close to ours, however, without considering the 3rd loop. Moreover, the functional relevance of these Pro residues was not discussed. Taking into account all the loops which constitute the membrane-binding motif of CTs, we suggest a new CT classification and believe that it is universal and can be used for a more precise analysis of the structure–function relationships in CTs. 

## 6. Role of Membrane Activity and Net Electrical Charge of CTs in Cytotoxicity 

Based on all of the above considerations, one may assume that in cases where the biological effect of several CTs is determined by their membrane activity, the effect will be more pronounced in those CTs that belong to the group with a higher number (Table 3). To illustrate this assumption, we need data on the cytotoxic activity of CTs from different groups. Unfortunately, not all studied sets of CTs meet this criterion. For example, in the study of Suzuki-Matsubara et al., there are five CTs from the venom of *N. naja*, but they belong exclusively to only two groups: five and eight [115]. This is not surprising since the distribution of CTs among the groups is uneven. The most numerous, group 5, contains 36 members (Table 4). Obviously, the reason for this is the simultaneous presence of the specific amino acid residues Ser28 and Asn45. However, their role can be discussed now only hypothetically (see next section). 

We can refer to our previously obtained data on cytotoxicities for five CTs (Table 2, CT3Nk, CT1No, CT1Nan, CT2No, and CT2Nan, belonging to groups 5, 5, 1, 8, and 3, respectively) [123]. It was established that in terms of the activity against HL60 and K562 cells, the toxins were arranged in correspondence with the increasing group number. However, CT3Nk from group 5 was found to be the most active against WEHI-3 cells. The cytotoxic effect is likely related to the action of CTs on lysosomes. Therefore, the activity depends on the number of CT molecules that have entered the lysosomes, not only on their membrane activity. The former may depend on the total electrical charge of a CT molecule. The activity of the seven CTs mentioned above (CT1Nn, CT7Nn, CT2Nk, CT2Nn, CT1Nan, CT2Nan, and newCT) against A549 cells also differs from their ability to cause dye leakage from liposomes [68]. 

It is clear that the ability of CTs to penetrate through bacterial peptidoglycan depends on the total charge of a CT molecule [124]. For example, when the antibacterial activity of five CTs (CT3Nk, CT1No, CT1Nan, CT2No, and CT2Nan from groups 5, 5, 1, 8, and 3, respectively) was studied against *M. luteus*, it was found that both CTs with two Pro residues in the loop-1 (CT1Nan and CT2Nan) and with one Pro residue in this loop (CT1No) have minimal activity if the total electrical charge is lower than seven [125]. In the case of *B. subtilis*, the activity of CLBP A5 (Table S2) is clearly higher than that of CT3Nk. This is precisely because of the higher electrical charge of CLBP A5 [126].

Let us now consider in more detail the relationships between the activities of CTs from the same group. In particular, let us focus on the most numerous, group 5 (Table 4). At this time, we cannot say with certainty which toxins within the group will be the most active. As can be seen from the above examples, the net electrical charge of a CT molecule is likely to be a determining factor. Thus, CTs with a higher charge will possess higher activity. A recent study of the cytotoxic activity of five CTs (CT1No, CT1Na, CT1Nan, CT2No, and CT2Nan, belonging to groups 5, 5, 1, 8, and 3, respectively) against rat cardiomyocytes is in line with this observation [119]. Four CTs (CT3Nk, CT1No, CT2bNsp, and CT2No from groups 5, 5, 8, and 8) were tested against the infusoria *Tetrahymena pyriformis* [127]. The activities of CTs from these series followed their group affiliation, and within the groups, the representative with a higher electrical charge exhibited a higher level of activity. 

## 7. What Are the Roles of Special Amino Acid Residues in the CT Activities?

What role do special amino acid residues in the loops of CTs play? To answer this question, two assumptions can be considered. First, all the special residues may be required to maintain the hydrophobicity gradient between the loops. Second, they may be required to interact with molecular targets other than lipids. The Pro9 residue is clearly not the first option. The first loop is distinguished from the other two by its maximal hydrophobicity. It differs significantly from the loop-1 of NTs as well (Figure 3a,c, residues 6–11). An alternative to Pro9 in loop-1 is the residue Ile/Leu for type IA CTs (Figure 3a) and Phe for type IB (Figure 3b). Therefore, it appears that it is the cis-bond between Pro8 and Pro9 residues that plays an important role. It may be necessary for interactions with some molecular targets, as noted earlier [21]. Therefore, option #2 is more preferable. Let us consider it in more detail. 

The spectrum of CT interactions is quite wide. Apart from lipids, other low-molecular-weight substances such as nucleotide triphosphates [128], citrate [129], and acenaphtene [130] can be mentioned. The list of high molecular biomolecules includes DNA [131,132], sugars [133,134,135,136], and proteins [137,138,139,140]. However, it is well established that the formation of CT/carbohydrate complexes occurs via clusters of lysine residues [134,141], most of which border the loops (Figure 3a,b). Proteins can be considered as one of the targets for binding, to which the presence of special residues in the CTs may be necessary. For example, the interaction of CTs with phospholipases A2 (PLA2) was demonstrated recently [139]. It has been shown that the strongest CT/PLA2 complexes are formed for CTs with two prolines in the first loop [139]. In venoms of the spitting cobra, it is the CTs with two Pro in loop-1 that predominate. This was clearly demonstrated by the quantitative analysis of the venoms of the following African cobras: *N. nigricollis*, *N. katensis*, *N. pallida*, *N. nubiae*, and *N. mossambica* [142]. In addition, the phospholipase A2 content is elevated in the venoms of spitting cobras [10]. In the venoms of non-spitting cobras, e.g., *N. atra*, CTs with a single Pro residue in loop-1 are prevalent [143]. This may indicate that these more membrane-active CTs do not require a synergism with PLA2 or need it to a lesser extent than CTs with two Pro in the loop-1. This assumption is consistent with the cytotoxicity of the venoms from a number of cobras [83]. The venom of Asian non-spitting cobras (*N. atra*, *N. kaouthia*, *N. naja*, and *N. oxiana*) and African spitting cobras (*N. mossambica*, *N. nigricollis*, and *N. pallida*) is approximately equally toxic. The venoms of African spitting cobras are dominated by CTs with two Pro residues in the first loop [142]. However, this is not true for Asian spitting cobras (*N. sumatrana*, *N. siamensis*, and *N. philippinensis*), whose venoms are dominated by CTs carrying a single Pro-reside in loop-1 [10]. This indicates that it is likely that CTs with either one or two Pro-residues in loop-1 can potentially form complexes with PLA2. Melittin, which is structurally disordered in aqueous solution [144], can form such a complex too [139], thus indicating the flexible structural requirements for such a complexation.

Usually, the multifunctionality of proteins, i.e., their capability to interact with multiple targets, is associated with their multidomain organization (e.g., [145]), their intrinsically disordered structure [146], or conformational flexibility [147]. Their multitargeted capability was already noted for linear cytolytic peptides, which are disordered in aqueous solution. Most likely, the membrane-binding motif of CTs, consisting of the three loops (Figure 1d), is capable of interacting with PLA2 and possibly other venom components, e.g., anticoagulant toxins [148], to synergize with them. Thus, CT multifunctionality is mediated via a single three-loop structural motif through grafting special amino acid residues into it.

## 8. Conclusions

The presence of the specific amino acid residues in CT loops 1–3 and their influence on the CT membrane activity allowed us to divide all known CTs into eight distinct groups. We classify the toxins containing a combination of Pro9, Ser28, and Asn/Asp45 residues as group 1, and toxins from this group possess the lowest membrane activity. CTs without any specific residues in the loops are combined into group 8, with the highest membrane activity. When only the membrane activity determines the CTs’ cytotoxic activity, the following hierarchy should be observed: the activity of CTs from a group with a higher number should exceed the activity of CTs from a group with a lower number. Thus, our classification can reliably predict the cytotoxic activity of CTs based on their amino acid sequences. The presence of specific amino acid residues in the three loops of CTs may indicate the involvement of these residues in the CTs’ interaction with non-lipid targets.

## Data Availability

Not applicable.

## Supplementary Materials

The following supporting information can be downloaded at: https://www.mdpi.com/article/10.3390/toxins16060262/s1, Figure S1: Aligned amino acid sequences of CLBP listed in Table S1. Table S1: Physico-chemical and bioinformatics data on three-finger cytotoxins (cardiotoxins). Table S2: Physico-chemical data and database codes for Cardiotoxin-like Basic Polypeptides (CLBPs) from Elapid venoms. Table S3: List of short neurotoxins used to build up the logo-sequence in Figure 3c. Table S4: Distribution of CTs across groups, determining their membrane activity.

## Abbreviations

CTs: cytotoxins; CLBPs, Cardiotoxin-like Basic Polypeptides; DOPC, dioleoyl-PC; DOPG, dioleoyl-PG; LD50, lethal dose resulting in death of 50% of tested animals; NT, short neurotoxins; PLA2, phospholipase A2; PC, phosphatidylcholine; PG, phosphatidylglycerol; PS, phosphatidylserine; TFT, Three-Finger Toxins.
