# Supplementary material for: Specific Amino Acid Residues in the Three Loops of Snake Cytotoxins Determine Their Membrane Activity and Provide a Rationale for a New Classification of These Toxins
Source: Toxins (Basel). 2024 Jun 4;16(6):262. doi: 10.3390/toxins16060262 (PMC11209149; doi:10.3390/toxins16060262)
Supplement: Supplementary file 1 [file toxins-16-00262-s001.zip › toxins-3012780-supplementary.pdf]

Supplemental info to the work:

Dubovskii, P.V.; Utkin, Y.N. Specific Amino Acid Residues in the Three Loops of Snake Cytotoxins Determine Their Membrane Activity and Provide a Rationale for a New Classification of These Toxins. *Toxins* **2024**, *16*, 262. https://doi.org/10.3390/toxins16060262.

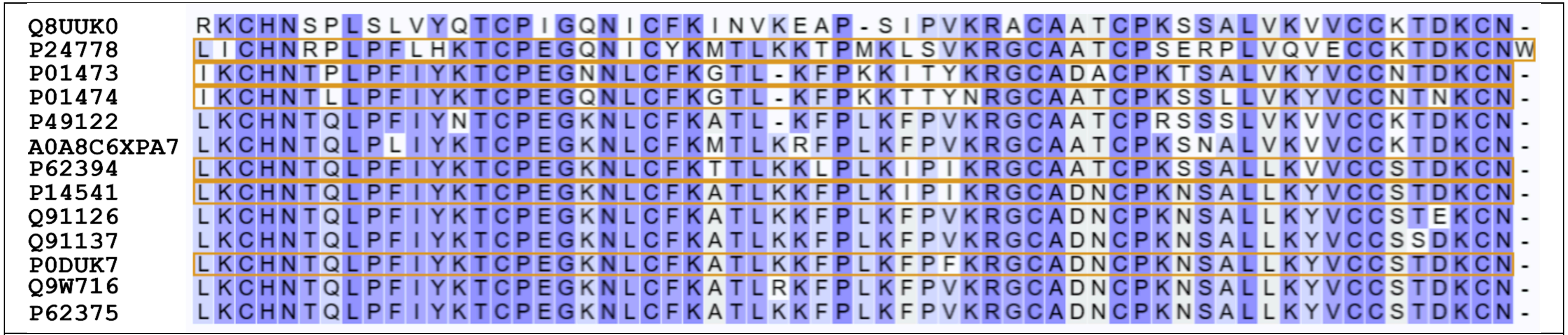

Figure S1. Aligned amino acid sequences of CLBP listed in Table S1. The program CLUSTAL O(1.2.4) for multiple sequence alignment was used.

Table S1. Physico-chemical and bioinformatics data on three-finger cytotoxins (cardiotoxins).

ID	Length	Molecular weight (Da)[1]	Charge[1] (pH 7.0)	Isoelectr. Point[1]	Group[2]	GenBank/ UniProt (Swiss-Prot)[3]	Snake	Name	Spatial structure/ Chemical shifts[4]	Similarity[5]
1	60	6791.11	6.45	8.80	1	None/ P01458	N. nivea	Cytotoxin 3	None	None
2	60	6802.3	9.45	9.63	1	None/ P01461	N. annulifera	Cytotoxin 4/ Toxin CM-11	None	None
3	60	6773.09	5.55	8.54	1	None/ P01457	N. haje	Cytotoxin 5/ Toxin CM-8	None	None
4	60	6839.27	6.45	8.78	1	None/ P01459	N. annulifera	Cytotoxin 5/ Toxin CM-8 (CM-8A)	None	None
5	60	6812.26	7.55	9.08	1	765048C/ P01460	N. annulifera	Cytotoxin 8/ Toxin CM-7	None	None
6	60	6696.09	6.55	8.8	1	731687A/ P01455	N. annulifera	Cytotoxin 1/ Toxin V(II)1	None	None
7	60	6697.08	5.55	8.55	1	None/ P01456	N. nivea	Cytotoxin 1/ Toxin V(II)1	None	None
8	60	6871.37	5.55	8.54	3	None/ P01463	N. nivea	Cytotoxin 2/ Toxin V(II)2	None	None
9	60	6856.4	7.55	9.08	3	770226C/ P01464	N. annulifera	Cytotoxin 5/ Toxin CM-6	None	None
10	60	6858.37	5.55	8.54	3	754240A/ P01462	N. annulifera	Cytotoxin 2/ Toxin V(II)2/ V(II)2A	None	None
11	60	6857.38	6.55	8.79	3	770226A/ P01465	N. annulifera	Cytotoxin 6/ Toxin CM-2H	None	None
12	60	6856.4	7.55	9.08	3	770226B/ P01466	N. annulifera	Cytotoxin 7/ Toxin CM-4B	None	None
13	61	6910.32	7.45	9.1	4	None/ Q98965	N. atra	Cytotoxin 6	None	None
14	60	6689.15	7.45	9.09	4	CAB42058/ CAA07687/ AAB86638/ P80245	N. atra	Cytotoxin (cardiotoxin) 6/ Cardiotoxin A6 (N)	1UG4/None	Three-finger toxin –N. atra -E2IU05; P49124; Q9PWQ7

15	60	6632.09	7.45	9.09	4	CAA90965/ P49123	N. atra	Cytotoxin (cardiotoxin) 8	None	None
16	60	6827.4	8.45	9.43	4	None/ P01468	N. pallida	Cytotoxin 1/ Cardiotoxin gamma	1CXO/None; 1CXN/None; 1TGX/None	None
17	60	6800.31	9.45	9.7	4	740421B/ P01469	N. mossambica	Cytotoxin 2/ Cardiotoxin 2A/ Cytotoxin V(II)2	None	None
18	60	6697.02	6.55	8.79	4	CAB41507/ Q9W6W6	N. atra	Cytotoxin (cardiotoxin) 10	None	None
19	60	6826.42	9.45	9.72	4	740421A/ P01467	N. mossambica	Cytotoxin 1/ Cardiotoxin IIB/ Cytotoxin V(II)1	2CCX/None	None
20	60	6894.47	9.45	9.72	4	740421C/ P01470	N. mossambica	Cytotoxin 3/ V(II)3	None	None
21	60	6895.45	8.45	9.43	4	None/P0DSN1	N. nigricollis	Naniproin	None	None
22	60	6764.12	6.45	8.78	5	AAB35381.1/None	N. naja	CLBP[6]	None	None
23	60	6723.23	7.55	9.12	5	CAA90962.1/ None	N. naja	Cardiotoxin 1	None	None
24	60	6701.18	6.45	8.81	5	AAB25732.1/ None	N. naja	Cardiotoxin 1	None	None
25	59	6724.17	5.45	8.54	5	AAB24495.1/ Q9PS33	N. oxiana	Cytotoxin Vc-1	None	None
26	60	6791.19	7.45	9.06	5	None/ P01447	N. naja	Cytotoxin 1	None	Q9PSN2
27	60	6792.18	6.45	8.78	5	None/ P86382	N. naja	Cytotoxin 7	None	None
28	60	6745.21	8.45	9.38	5	None/ P24780	N. naja	Cytotoxin 3	None	None
29	60	6745.21	8.45	9.38	5	None/ P01445	N. kaouthia	Cytotoxin 2	7QFC, 7QHI, 7O2K/ 27877	A0A8C6XLA1_N. Naja; Uncharacterized protein; Cytotoxin 13 N. naja;
30	60	6717.16	8.45	9.38	5	None/ P01446	N. kaouthia	Cytotoxin 3	6RC7/ 34392	None
31	60	6807.28	8.45	9.38	5	None/ P0CH80	N. kaouthia	Cytotoxin 1	None	None
32	60	6792.29	9.45	9.63	5	None/ P60306	N. atra	Cytotoxin SP13b	None	None
33	60	6795.29	8.45	9.38	5	None/ Q98961	N. atra	Cytotoxin 5	None	None

34	60	6750.18	7.45	9.09	5	AAB18377.1 (AAB18381.1; CAB42054.1)/ P01442	N. atra	Cytotoxin 2/ Cardiotoxin 1A/ Cardiotoxin 2/ Cardiotoxin A2/ Cardiotoxin II/ Cardiotoxin analog II	1CRE, 1CRF/ None; 4OM4/None	Cytotoxin 5a N. sputatrix-AAC27691.1/AAC61317.1/O73857; Cytotoxin 5b N. sputatrix-P60310
35	60	6821.28	5.45	8.54	5	PRF: 229587/ P01451	N. oxiana	Cytotoxin I	1RL5, 5NPN/ 5989; 1ZAD/None; 5NQ4/ 34125;	None
36	60	6701.18	6.45	8.81	5	AAB01539.1/ P60304	N. atra	Cytotoxin 1/ Cardiotoxin 1/ Cardiotoxin A1/ Cardiotoxin I/ Cardiotoxin analog I	2CDX/ None	Cytotoxin 1, N. kaouthia - P60305
37	60	6701.13	5.45	8.55	5	AAB18380/ Q98958	N. atra	Cytotoxin (cardiotoxin) 1d/1e	None	None
38	60	6725.16	6.55	8.81	5	AAB36927/ P79810	N. atra	Cytotoxin (cardiotoxin) 1c	None	None
39	60	6704.2	5.45	8.55	5	CAA63978/ Q91135	N. atra	Cytotoxin (cardiotoxin) I –like P-15	None	None
40	60	6685.13	6.45	8.81	5	AAB18379/ Q98957	N. atra	Cytotoxin (cardiotoxin) 1a	None	None
41	60	6705.16	6.45	8.81	5	CAA63977/ Q91136	N. atra	Cytotoxin (carditoxin) I-like T-15	None	None
42	60	6715.25	9.45	9.63	5	None/ P01452	N. mossambica	Cytotoxin (cardiotoxin) 4/V(II)	1CDT/None	None
43	60	6668.94	3.56	8.12	5	765048A/ P01454	N. annulifera	Cytotoxin 9/ Toxin CM-2e	None	None

44	60	6681.94	3.56	8.12	5	765048B/ P01453	N. annulifera	Cytotoxin 10/ Toxin CM-4a	None	None
45	60	6793.21	8.45	9.4	5	AAB86637, CAA73098, CAA73096/ P01443	N. atra	Cytotoxin 4/ Cardiotoxin A4/ Cardiotoxin analog IV	1KBT, 1KBS/ None; 4OM5/None	None
46	60	6765.2	8.45	9.38	5	None/ O73858	N. sputatrix	Cytotoxin (cardiotoxin) 6	None	None
47	60	6797.26	8.45	9.38	5	AAC61319/ O73859	N. sputatrix	Cytotoxin (cardiotoxin) 7	None	None
48	60	6753.1	5.45	8.54	5	None/ P60311	N. sputatrix	Cytotoxin KJC3	None	None
49	60	6793.16	5.45	8.53	5	None/ P86540	N. naja	Cytotoxin 8	None	None
50	60	6775.15	7.45	9.06	5	None/A0A0U5AL91	N. naja	Cytotoxin 17	None	None
51	60	6791.19	7.45	9.06	5	BAU24671/ A0A8C6XG05	N. naja	Cytotoxin 14/ Uncharacterized protein	None	A0A8C7DYP2
52	60	6711.06	4.46	8.33	5	None/P86538	N. naja	Cytotoxin 2a	None	None
53	60	6765.16	7.45	9.06	5	None/A0A0U4W6K7	N. naja	Cytotoxin 16	None	
54	60	6761.21	8.45	9.34	5	None/A0A8C6XQ33	N. naja	Uncharacterized protein	None	None
55	60	6681.08	5.45	8.55	5	BAU24666/ A0A0U5ARS4	N. naja	Cytotoxin 11	None	None
56	60	6791.15	6.45	8.78	5	None/A0A0U5AR60	N. naja	Cytotoxin 12	None	None
57	60	6747.18	8.45	9.34	5	None/A0A0U4W6H0	N. naja	Cytotoxin 15	None	None
58	60	6810.35	8.45	9.38	6	None/ P07525	N. atra	Cytotoxin 5/ Cardiotoxin A4b/ Cardiotoxin T/ Cardiotoxin analog V/ Cytotoxin D-1/ Membrane toxin D1	1CHV/None	None
59	60	6603.07	5.45	8.55	6	AAB18378/ Q98956	N. atra	Cytotoxin (cardiotoxin) 1b	None	None

60	60	6682.02	5.55	8.55	6	740419A/ P01448	N. melanoleuca	Cytotoxin 1/V(II)1	None	None
61	60	6808.27	8.45	9.4	6	CAB42057/ Q9W6W9	N. atra	Cytotoxin (cardiotoxin) 4N	None	None
62	60	6780.25	6.45	8.79	6	None/A0A8C6XFH6	N. naja	Uncharacterized protein	None	None
63	60	6654.05	5.45	8.55	6	None/A0A8C6XFM5	N. naja	Uncharacterized protein	None	None
64	60	6654.14	8.45	9.38	7	None/ P24779	N. kaouthia	Cytotoxin 5	None	None
65	60	6774.29	8.45	9.38	7	CAB42053 (AAB18383)/ Q98959	N. atra	Cytotoxin 3a/ Cardiotoxin-31/ Cardiotoxin 3a	None	None
66	60	6636.13	8.45	9.43	7	AAB18386/ Q98962	N. atra	Cytotoxin (cardiotoxin) 3d	None	None
67	60	6732.21	8.45	9.38	7	AAB18384/ Q98960	N. atra	Cytotoxin (cardiotoxin) 3b	None	None
68	60	6714.17	8.45	9.38	7	AAG02235/ Q9DGH9	N. kaouthia	Cytotoxin 2	None	None
69	60	6790.29	9.45	9.58	7	-	N. naja	newCT	None	None
70	60	6747.27	8.45	9.38	8	AAA49386.1/ Q02454	N. sputartix	Cytotoxin	1XT3/None; 2BHI/None; 1H0J, 1I02, 2CRT, 2CRS/4966, 15305	Cytotoxin 3 N. sputatrix-P60302; cytotoxin 3 N. atra-P60301; cytotoxin 4 N. kaouthia-P60303; P01444; Q9PS23; Q9W6W7
71	60	6683.27	9.45	9.68	8	None/ P60307	N. atra	Cytotoxin SP15a	None	None
72	60	6802.31	9.45	9.56	6	None/ P83345	N. sagittifera	Cytotoxin sagitoxin	None	None
73	60	6763.27	8.45	9.34	8	None/ P01440	N. naja	Cytotoxin 2/ Cobramine-B/ Cytotoxin II/ Cytotoxin-9	None	None

74	60	6763.27	8.45	9.34	8	AAC27686/ Q9PST3	N. sputatrix	Cytotoxin (cardiotoxin) 2b	None	Cytotoxin 2a – N. sputatrix-Q9PST4; cytotoxin 9, N. naja - A0A0U5AUY6; cytotoxin 1, N. sumatrana - A0A7T7DMY7
75	60	6793.36	8.45	9.34	8	AAC27690/ O73856	N. sputatrix	Cytotoxin (cardiotoxin) 4b	None	None
76	60	6609.1	8.45	9.43	8	AAB01542/ Q91124	N. atra	Cytotoxin (carditoxin) 8/VIII	None	None
77	60	6777.36	8.45	9.38	8	AAC27689/ O93473	N. sputatrix	Cytotoxin (cardiotoxin) 4a	None	None
78	60	6729.23	8.45	9.38	8	AAC27684/ O93471	N. sputatrix	Cytotoxin (cardiotoxin) 1	None	None
79	60	6664.18	8.55	9.44	8	AAB24494/ Q9PS34	N. oxiana	Cytotoxin Vc-5	None	None
80	60	6636.17	8.55	9.42	8	None/ P01441	N. oxiana	Cytotoxin 2 (II)	1CCQ/4418,4419 1CB9/4419, 1FFJ/4815	None
81	60	6652.26	10.45	9.9	8	None/ P60309	N. atra	Cytotoxin SP15d	None	None
82	60	6755.33	8.45	9.4	8	None/ P60308	N. atra	Cytotoxin SP15c	None	None
83	60	6845.33	7.46	9.1	8	None/ P25517	N. mossambica	Cytotoxin 5	None	None
84	60	6543.09	10.45	9.9	8	None/ E2ITZ7	N. atra	Three-finger toxin	None	None
85	61	6844.45	9.55	9.72	8	None/ P01471	Hemachatus haemachatus	Cytotoxin 1/ hemolytic protein 12B	None	None

86	61	6844.45	9.55	9.72	8	None/ B3EWH9	Hemachatus haemachatus	Three-finger hemachatoxin	3VTS/None	None
87	61	6793.27	6.55	8.81	8	None/ P24777	Hemachatus haemachatus	Cytotoxin 3/ Toxin 11(11A)	None	None
88	61	6800.33	9.55	9.72	8	None/ P24776	Hemachatus haemachatus	Cytotoxin 2/ Toxin 12A	None	None
89	60	6764.25	7.45	9.06	8	None/ P86541	N. naja	Cytotoxin 10	None	None
90	60	6823.3	8.52	9.53	8	None/O93472	N. sputatrix	Cytotoxin 2c	None	None

[1] - Molecular weight, electrical charge and isoelectric point were calculated using program modlamp (https://github.com/alexarnimueller/modlAMP);

[2] - The groups are listed in the separate Table, according to the special amino acid residues within the three loops;

[3] - Databank identifiers;

[4] - PDB-code in the PDB-bank (www.rcsb.org)/ chemical shifts in the Biological Magnetic Resonance Databank (BMRB, http://www.bmrb.wisc.edu/);

[5] - Identical polypeptides – Name/Snake/SwissProte identifier;

[6] - CLBP, Cardiotoxin-Like Basic Polypeptide. Here, the term "cytotoxin" is more relevant.

Table S2. Physico-chemical data and database codes for Cardiotoxin-like Basic Polypeptides (CLBP) from Elapid venoms.[1]

Id	Length[2]	M.W.(Da)[2]	Charge[2] (pH 7.0)	Isoelectr. point	Uniprot/NLM[3] code	Snake	Name	Similarity
1	61	6608.89	7.55	9.15	sp|Q8UUK0|3SOF3_NAJAT	N. atra	Cytotoxin homolog Clbp-3	None
2	63	7192.62	6.66	8.82	sp|P24778|3SOE_HEMHA	Hemachatus haemachatus	Cytotoxin homolog	None
3	61	6833.1	7.55	9.07	sp|P01473|3SOF3_NAJME	N. melanoleuca	Cytotoxin homolog 3	None
4	61	6850.13	8.55	9.38	sp|P01474|3SOF2_NAJME	N. melanoleuca	Cytotoxin homolog 2	None
5	61	6795.14	8.55	9.5	sp|P49122|3SOF7_NAJAT	N. atra	Cytotoxin 7	None
6	62	6974.51	10.55	9.98	tr|A0A8C6XPA7|A0A8C6XPA7_NAJNA	N. naja	Snake toxin/toxin-like domain-containing protein	None
7	62	6842.33	9.55	9.75	sp|P62394|3SOFB_NAJHH	N. haje haje	Cytotoxin 11	P62390
8	62	6994.39	8.55	9.4	sp|P14541|3SOFH_NAJKA	N. kaouthia	Cytotoxin homolog	None
9	62	7028.41	8.55	9.4	sp|Q91126|3SOFA_NAJAT	N. atra	Cardiotoxin 7a	None
10	62	7000.36	8.55	9.4	sp|Q91137|3SOFL_NAJAT	N. atra	Cytotoxin homolog 5	None
11	62	7062.43	8.55	9.4	sp|P0DUK7|3SOF1_NAJAT	N. atra	Mu-elapitoxin-Na1a	None
12	62	7042.39	8.55	9.42	sp|Q9W716|3SOFV_NAJAT	N. atra	Cytotoxin homolog 5V	None
13	62	7014.38	8.55	9.4	sp|P62375|3SOF5_NAJAT	N. atra	Cytotoxin A5	P62377 Q91996 A0A8C6XRN7

[1] Aligned amino acid sequences of the toxins (without signal peptides) are given in Figure S1.

[2] Molecular weight, electrical charge and isoelectric point were calculated using program modlamp (https://github.com/alexarnimueller/modlAMP);

[3] NLM, US National Library of Medicine (https://www.ncbi.nlm.nih.gov/).

Table S3. List of short neurotoxins used to build up the logo-sequence in Figure 3c.

<table>
<tr><th>No</th><th>Identificator</th><th>Amino acid sequence[1]</th></tr>
<tr><td>1</td><td>sp|C1IC48|3S14_</td><td>LLCHNQQSSTSPTTTCCSGGESKCYKKRWPTHRGTITERGCGCPTVKKGIELHCCTTDQCNL</td></tr>
<tr><td>2</td><td>sp|P01432|3S13_</td><td>LNCHNQMSAQPPTTTRCSRWETNCYKKRWRDHRGYKTERGCGCPTVKKGIQLHCCTSDNCNN</td></tr>
<tr><td>3</td><td>sp|P34076|3S11_</td><td>MECHNQQSSQPPTTTHCSGGETNCYEKRWHDHRGTIIERGCGCPTVKPGVKLNCCTTDKCNN</td></tr>
<tr><td>4</td><td>sp|C0HM08|3S12_</td><td>LECHNQQSSQTPTTTDCSGGETNCYKKWWSDHRGTIIERGCGCPTVKKGIELNCCTTDRCNN</td></tr>
<tr><td>5</td><td>tr|A0A8C6XDY8|A</td><td>LECHNQQSSQPPTTTCC-SGETNCYKKRWRDHRGYRTERGCGCPSVRNGIEINCCTTDRCNN</td></tr>
<tr><td>6</td><td>sp|P82849|3S1B2</td><td>LECHNQQSSQTPTTTGCSGGENNCYKKEWRDNRGYRTERGCGCPSVKKGIGINCCTTDRCNN</td></tr>
<tr><td>7</td><td>sp|P01431|3S11_</td><td>LECHNQQSSEPPTTTRCSGGETNCYKKRWRDHRGYRTERGCGCPTVKKGIELNCCTTDRCNN</td></tr>
<tr><td>8</td><td>sp|O57327|3S1A4</td><td>LECHNQQSSQAPTTTGCSGGETNCYKKGWRDHRGYRIERGCGCPSVKKGIEINCCTTDRCNN</td></tr>
<tr><td>9</td><td>sp|Q9YGJ5|3S1A2</td><td>LECHNQQSSQAPTTTGCSGGETNCYKKSWRDHRGYRIERGCGCPSVKKGIEINCCTTDRCNN</td></tr>
<tr><td>10</td><td>sp|O57326|3S1A3</td><td>LECHNQQSSETPTTTGCSGGETNCYKKRWRDHRGYRIERGCGCPSVKKGIEINCCTTDRCNN</td></tr>
<tr><td>11</td><td>sp|Q9YGJ6|3S1A1</td><td>LECHNQQSSETPTTTGCSGGETNCYKKSWRDHRGYRIERGCGCPSVKKGIEINCCTTDRCNN</td></tr>
<tr><td>12</td><td>sp|E2ITZ3|3S11A</td><td>LECHNQQSSQTPTTTGCSGGETNCYKKRWRDHRGYRTERGCGCPSVQTAIEINCCTTDRCNN</td></tr>
<tr><td>13</td><td>sp|P14613|3S1B1</td><td>LECHNQQSIQTPTTTGCSGGETNCYKKRWRDHRGYRTERGCGCPSVKNGIEINCCTTDRCNN</td></tr>
<tr><td>14</td><td>sp|Q9PTT0|3S1CB</td><td>LECHNQQSSQTPTTTGCSGGETNCYKKRWRDHRGYRTERGCGCPSVKNGIEINCCTTDRCNN</td></tr>
<tr><td>15</td><td>sp|Q9PSN6|3S13_</td><td>LECHDQQSSQTPTTTGCSGGETNCYKKRWRDHRGYRTERGCGCPSVKNGIEINCCTTDRCNN</td></tr>
<tr><td>16</td><td>sp|P01420|3S13_</td><td>MICYKQQSLQFPITTVC-PGEKNCYKKQWSGHRGTIIERGCGCPSVKKGIEINCCTTDKCNR</td></tr>
<tr><td>17</td><td>sp|P01421|3S14_</td><td>MICYKQRSLQFPITTVC-PGEKNCYKKQWSGHRGTIIERGCGCPSVKKGIEINCCTTDKCNR</td></tr>
<tr><td>18</td><td>sp|P34075|3S11_</td><td>KICYNQPSSQHPTTKAC-PGEKNCYRKQWSDHRGTIIERGCGCPTVKPGVKLHCCTTEKCNN</td></tr>
<tr><td>19</td><td>sp|P01433|3S12_</td><td>LECHNQQSSQTPTTQTC-PGETNCYKKQWSDHRGSRTERGCGCPTVKPGIKLKCCTTDRCNK</td></tr>
<tr><td>20</td><td>sp|P25675|3S12_</td><td>MICHNQQSSQPPTIKTC-PGETNCYKKQWRDHRGTIIERGCGCPSVKKGVGIYCCKTDKCNR</td></tr>
<tr><td>21</td><td>sp|P01422|3S12_</td><td>MICHNQQSSQPPTIKTC-PGETNCYKKRWRDHRGTIIERGCGCPSVKKGVGIYCCKTNKCNR</td></tr>
<tr><td>22</td><td>sp|P01423|3S12_</td><td>MICHNQQSSQRPTIKTC-PGETNCYKKRWRDHRGTIIERGCGCPSVKKGVGIYCCKTDKCNR</td></tr>
<tr><td>23</td><td>sp|P68417|3S11_</td><td>LECHNQQSSQPPTTKTC-PGETNCYKKRWRDHRGSITERGCGCPSVKKGIEINCCTTDKCNN</td></tr>
<tr><td>24</td><td>sp|P01425|3S11_</td><td>LECHNQQSSQPPTTKSC-PGDTNCYNKRWRDHRGTIIERGCGCPTVKPGINLKCCTTDRCNN</td></tr>
<tr><td>25</td><td>sp|P01424|3S11_</td><td>MECHNQQSSQPPTTKTC-PGETNCYKKQWSDHRGTIIERGCGCPSVKKGVKINCCTTDRCNN</td></tr>
<tr><td>26</td><td>sp|P01426|3S11_</td><td>LECHNQQSSQPPTTKTC-PGETNCYKKVWRDHRGTIIERGCGCPTVKPGIKLNCCTTDKCNN</td></tr>
<tr><td>27</td><td>sp|P80958|3S1CC</td><td>LECHNQQSSQTPTTKTC-SGETNCYKKWWSDHRGTIIERGCGCPKVKPGVNLNCCTTDRCNN</td></tr>
<tr><td>28</td><td>sp|P59275|3S1B_</td><td>LECHNQQSSQTPTTKTC-SGETNCYKKWWSDHRGTIIERGCGCPKVKPGVNLNCCRRDRCNN</td></tr>
<tr><td>29</td><td>sp|P60774|3S11_</td><td>LECHNQQSSQAPTTKTC-SGETNCYKKWWSDHRGTIIERGCGCPKVKPGVKLNCCTTDRCNN</td></tr>
<tr><td>30</td><td>sp|P60773|3S11_</td><td>LECHNQQSSQAPTTKTC-SGETNCYKKWWSDHRGTIIERGCGCPKVKPGVKLNCCRTDRCNN</td></tr>
<tr><td>31</td><td>sp|P01427|3S11_</td><td>LECHNQQSSQPPTTKTC-SGETNCYKKWWSDHRGTIIERGCGCPKVKPGVNLNCCRTDRCNN</td></tr>
<tr><td></td><td>sp|P59276|3S1C_</td><td>LECHNQQSSQAPTTKTC-SGETNCYKKWWSDHRGTIIERGCGCPKVKPGVNLNCCRTDRCNN</td></tr>
</table>

32	sp	P01416	3S11_	RICYNHQSTTRATTKSCE--ENSCYKKYWRDHRGTIIERGCGCPKVKPGVGIHCCQSDKCNY
33	sp	P01418	3S11_	RICYNHQSTTPATTKSCG--ENSCYKKTWSDHRGTIIERGCGCPKVKRGVHLHCCQSDKCNN
34	sp	P01417	3S11_	RICYNHQSTTPATTKSCG--ENSCYKKTWSDHRGTIIERGCGCPKVKQGIHLHCCQSDKCNN
35	sp	F5CPD8	3S157	MICYNQQSSQPPTTTTCS--EGQCYKQRWRDHRGWRTERGCGCPKAIPEVKLNCCKTDRCNG
36	sp	P80548	3S11_	MICHNQQSSQPPTIKTCS--EGQCYKKTWRDHRGTISERGCGCPTVKPGIHISCCASDKCNA
37	sp	K9MCH1	3S11_	RICYNQQSSQPPTTKTCS--EGQCYKKTWRDHRGTIIERGCACPNVKPGIQISCCTSDKCNG
38	sp	P86420	3S11_	MICYNHQSSEPPTTKTCS--EGQCYKKSWSDHRGTIIERGCACPNVKPGVKIICCRSC----
39	sp	P0CAR1	3S1D1	MICYNQQSSQPPTTKTCS--EGQCYKKTWSDHRGTISERGCACPNVKPGVKISCCSSDKCNG
40	sp	F5CPD5	3S151	MICYNQQSSQPPTTTTCS--EGQCYKKTWSDHRGTIIERGCACPNVKPGVKISCCSSDKCNG
41	sp	P86095	3S11_	MICYNQQSTEPPTTKTCS--EGQCYKKTWSDHRGTIIERGCACPNVKPGVKISCCSSDKCR-
42	sp	C0HLK3	3S11_	MICYNQQSSEPPTTKTCS--EGQCYKKTWSDHRGTIIERGCACPNVKPGVKISCCSSDK---
43	sp	Q7T2I5	3S1EC	RICFNHQSSQPQTTKTCSPGESSCYHKQWSDFRGTIIERGCGCPTVKPGINLSCCESEVCNN
44	sp	P10457	3S12_	RRCYNQQSSQPKTTKSCPPGENSCYNKQWRDHRGSITERGCGCPTVKPGIKLRCCESEDCNN
45	sp	Q9YGC7	3S15_	RRCFNQQSSQPKTTKSCPLGENSCYNKQWRDHRGSITERGCGCPKVKPGIKLRCCESEDCNN
46	sp	Q9YGX0	3S14_	RRCFNQQSSQPKTTKSCPLGENSCYNKQWRDHRGSITERGCGCPKVKPGIKLRCCESEDCNN
47	sp	P10455	3S1C_	RRCYNQQSSQPKTTKSCPPGENSCYNKQWRDHRGSITERGCGCPKVKPGIKLRCCESEDCNN
48	sp	P10456	3S1D_	RRCFNQQSSQPKTTKSCPPGENSCYNKQWRDHRGSITERGCGCPKVKPGIKLRCCESEDCNN
49	sp	P10460	3S1C_	RRCFNQQSSQPQTNKSCPPGENSCYRKQWRDHRGTIIERGCGCPTVKPGVKLRCCQSEDCNN
50	sp	Q7T2I1	3S110	RRCFNQQSSEPQTNKSCPPGENSCYRKQWRDHRGTIIERGCGCPTVKPGVKLRCCESEDCNN
51	sp	Q9YGX1	3S1ED	RRCFNQQSSEPQTNKSCPPGENSCYRKQWRDHRGTIIERGCGCPTVKPGIKLRCCESEDCNN
52	sp	Q9YGW8	3S14_	RRCFNQQSSEPQTNKSCPPGENSCYNKQWRDHRGTIIERGCGCPQVKSGIKLTCCQSDDCNN
53	sp	Q9YGW9	3S12_	RRCFNQQSSEPQTNKSCPPGENSCYNKQWRDHRGTITERGCGCPQVKSGIKLTCCQSDDCNN
54	sp	P25496	3S1B_	RRCFNHPSSQPQTNKSCPPGENSCYNKQWRDHRGTIIERGCGCPQVKSGIKLRCCQSDDCNN
55	sp	P25495	3S1A_	RRCFNHPSSQPQTNKSCPPGENSCYNKQWRDHRGTIIERGCGCPTVKPGIKLRCCQSDDCNN
56	sp	Q9YGC2	3S11_	RRCFNHPSSQPQTNKSCPPGENSCYNKQWRDHRGTIIERGCGCPTVKPGIKLTCCQSEDCNN
57	sp	P19959	3S12_	LTCCNQQSSQPKTTTDCA--DNSCYKMTWRDHRGTRIERGCGCPQVKPGIKLECCKTNECNN
58	sp	P19960	3S14_	LTCCNQQSSQPKTTTDCA--DDSCYKKTWKDHRGTRIERGCGCPQVKPGIKLECCKTNECNN
59	sp	P32879	3S11_	LTCCNQQSSQPKTTTDCA--DNSCYKKTWQDHRGTRIERGCGCPQVKPGIKLECCKTNECNN
60	sp	P19958	3S13_	LTCCNQQSSQPKTTTDCA--DNSCYKKTWKDHRGTRIERGCGCPQVKPGIKLECCKTNECNN
61	sp	Q5UFR8	3S11_	MTCCNQQSSQPKTTTNCA--GNSCYKKTWSDHRGTIIERGCGCPQVKSGIKLECCHTNECNN
62	sp	P62388	3S1A_	MTCCNQQSSQPKTTTNCA--ESSCYKKTWSDHRGTRIERGCGCPQVKSGIKLECCHTNECNN
63	sp	P25494	3S11_	MTCCNQQSSQPKTTTNCA--ESSCYKKTWSDHRGTRIERGCGCPQVKKGIKLECCHTNECNN
64	sp	Q8UW27	3S116	MTCCNQQSSQPKTTTNCA--ESSCYKKTWSDHRGTRIERGCGCPQVKRGIKLECCHTNECNN
65	sp	Q8UW26	3S12_	MTCCNQQSSQPKTTTNCA--ESSCYKKTWSDHRGTRIERGCGCPQVKHGIKLECCHTNECNN
	sp	A8HDJ8	3S12_	MTCCNQQSSQPKTITTCA--ESSCYKKTWKDHHGTRIERGCGCPKVKPGVGLECCKTDECNN

<table>
<tr><td>66</td><td>sp|F8J2G3|3S12A</td><td>MTCYNQQSSQPQTTTTCA--ESSCYKKTWRDHRGTIIERGCGCPTVKPGIQRVCCATDKCNN</td></tr>
<tr><td>67</td><td>sp|A8HDJ6|3S12_</td><td>MTCCNQQSSQPKTTTPCA--ESSCYKKTWKDHRGTIIERGCGCPNVKPGIDLMCCRTDECNN</td></tr>
<tr><td>68</td><td>sp|A8HDJ5|3S11_</td><td>MTCCNQQSSQPKTTTPCA--ESSCYKKTWKDNRGTIIERGCGCPNVKPGIDLMCCKTDECNN</td></tr>
<tr><td>69</td><td>sp|A8HDJ9|3S11_</td><td>MTCCNQQSSQPKTTKTCA--ESSCYKKTWRDHRGTITERGCGCPSVKPGIQLECCKTNECNN</td></tr>
<tr><td>70</td><td>sp|A8S6A4|3S11_</td><td>MTCCNQQSSQPKTTTTCA--ESSCYKKTWRDHRGTIIERGCGCPNVKPGIQLVCCETNECNN</td></tr>
<tr><td>71</td><td>sp|A7X4R5|3S13_</td><td>MTCYNQQSSQAKTTTTCSGGVSSCYRKTWSDIRGTIIERGCGCPSVKKGIERICCGTDKCNN</td></tr>
<tr><td>72</td><td>sp|P0CB06|3S12_</td><td>MTCYNQQSSEAKTTTTCSGGVSSCYKKTWSDIRGTIIERGCGCPSVKKGIERICCRTDKCNN</td></tr>
<tr><td>73</td><td>sp|Q45Z11|3S11_</td><td>MTCYNQQSSEAKTTTTCSGGVSSCYKKTWSDGRGTIIERGCGCPSVKKGIERICCRTDKCNN</td></tr>
<tr><td>74</td><td>sp|P01434|3S11_</td><td>MQCCNQQSSQPKTTTTCPGGVSSCYKKTWRDHRGTIIERGCGCPRVKPGIRLICCKTDECNN</td></tr>
<tr><td>75</td><td>sp|A8HDJ4|3S11_</td><td>MTCCNQQSSQPKTTTTCAGGESSCYKKTWSDHRGSRTERGCGCPHVKPGIKLTCCETDECNN</td></tr>
</table>

[1] – Aligned with CLUSTAL W (1.83) multiple sequence alignment.

Table S4. Distribution of CT across groups, determining their membrane activity[1].

Group	Amino acid sequence	Database identifier	ID # in Table S1
1, PSX	LKCNQLIP**P**FWKTCPKGKNLCYNMYMV**S**TSTVPVKRGCIDVCPK**N**SALVKYVCCNTDRCN	P01458	1
	LKCNKLIP**P**FWKTCPKGKNLCYKMYMV**S**TLTVPVKRGCIDVCPK**N**SALVKYVCCNTNKCN	P01461	2
	LKCHQLVP**P**FWKTCPEGKNLCYKMYMV**S**SSTVPVKRGCIDVCPK**N**SALVKYVCCNTDKCN	P01457	3
	LKCYKLVP**P**FWKTCPEGKNLCYKMYMV**S**TLTVPVKRGCIDVCPK**N**SALVKYVCCNTDKCN	P01459	4
	LKCHKLVP**P**FWKTCPEGKNLCYKMYMV**S**TLTVPVKRGCIDVCPK**N**SALVKYVCCNTNKCN	P01460	5
	LKCHKLVP**P**VWKTCPEGKNLCYKMFMV**S**TSTVPVKRGCIDVCPK**N**SALVKYVCCSTDKCN	P01455	6
	LKCHKLVP**P**VWKTCPEGKNLCYKMFMV**S**TSTVPVKRGCIDVCPK**D**SALVKYVCCSTDKCN	P01456	7
	In total: 7 items		
2, PSO	This group is empty		
	In total: 0 items		
3, POX	LKCHQLIP**P**FWKTCPEGKNLCYKMYMVATPMIPVKRGCIDVCPK**N**SALVKYMCCNTDKCN	P01463	8
	LKCHKLVP**P**FWKTCPEGKNLCYKMYMVATPMIPVKRGCIDVCPK**N**SALVKYMCCNTNKCN	P01464	9
	LKCHKLVP**P**FWKTCPEGKNLCYKMYMVATPMLPVKRGCIDVCPK**D**SALVKYMCCNTDKCN	P01462	10
	LKCHKLVP**P**FWKTCPEGKNLCYKMYMVATPMLPVKRGCIDVCPK**D**SALVKYMCCNTNKCN	P01465	11
	LKCHKLVP**P**FWKTCPEGKNLCYKMYMVATPMLPVKRGCINVCPK**D**SALVKYMCCNTNKCN	P01466	12
	In total: 5 items		
4, POO	LKCNQLIP**P**FYKTCAAGKNLCYKMFMVAAQRFPVKRGCIDVCPKSSLLVKYVCCNTDRCNN	Q98965	13
	LKCNQLIP**P**FYKTCAAGKNLCYKMFMVAAPKVPVKRGCIDVCPKSSLLVKYVCCNTDRCN	P80245	14
	LKCNQLIP**P**FYKACAAGKNLCYKMFMVAAPKVPVKRGCIDVCPKSSLLVKYVCCNTDRCS	P49123	15
	LKCNQLIP**P**FWKTCPKGKNLCYKMTMRAAPMVPVKRGCIDVCPKSSLLIKYMCCNTDKCN	P01468	16
	LKCNQLIP**P**FWKTCPKGKNLCYKMTMRGASKVPVKRGCIDVCPKSSLLIKYMCCNTDKCN	P01469	17
	LKCNQHIP**P**FYKTCAAGKNLCYKIFMVAAPKVPVKRGCIDVCPKSSDLVKYVCCNTDRCN	Q9W6W6	18
	LKCNQLIP**P**FWKTCPKGKNLCYKMTMRAAPMVPVKRGCIDVCPKSSLLIKYMCCNTNKCN	P01467	19
	LKCNRLIP**P**FWKTCPEGKNLCYKMTMRLAPKVPVKRGCIDVCPKSSLLIKYMCCNTNKCN	P01470	20

	LKCNRLIP**P**FWKTCPEGKNLCYKMTMRLAPKVPVKRGCIDVCPKSSLLIKYMCCTNDKCN<br>In total: 9 items	P0DSN1	21
5,<br>OSX	LKCNKLIPLAYKTCPAGKNLCYKMYMV**S**NKTVPVKRGCIDVCPK**N**SLLVTYECCNTDRCN	AAB35381.1	22
	LKCNKLIPIASKTCPAGKNLCYKMFMM**S**DLTIPVKRGCIDVCPK**N**SLLVKYVCCNTHRCN	CAA90962.1	23
	LKCNKLIPIASKTCPAGKNLCYKMFMM**S**DLTIPVKRGCIDVCPKS**N**LLVKYVCCNTDRCN	AAB25732.1	24
	LKCNKLVPIAYKTCE-GKNLCYKMFMM**S**DLTIPVKRGCIDVCPK**N**SLLVKYVCCNTDRCN	Q9PS33	25
	LKCNKLIPLAYKTCPAGKNLCYKMYMV**S**NKTVPVKRGCIDVCPK**N**SLVLKYECCNTDRCN	P01447	26
	LKCNKLIPLAYKTCPAGKDLCYKMYMV**S**NKTVPVKRGCIDVCPK**N**SLLVKYECCNTDRCN	P86382	27
	LKCNKLIPLAYKTCPAGKNLCYKMFMV**S**NKTVPVKRGCIDVCPK**N**SLVLKYVCCNTDRCN	P24780	28
	LKCNKLIPLAYKTCPAGKNLCYKMFMV**S**NKTVPVKRGCIDVCPK**N**SLLVKYVCCNTDRCN	P01445	29
	LKCNKLIPLAYKTCPAGKNLCYKMFMV**S**NKTVPVKRGCIDACPK**N**SLLVKYVCCNTDRCN	P01446	30
	LKCNKLVPLFYKTCPAGKNLCYKMFMV**S**NKTVPVKRGCIDVCPK**N**SLVLKYVCCNTDRCN	P0CH80	31
	LKCNKLKPLAYKTCPAGKNLCYKMFMM**S**NKTVPVKRGCIDVCPK**N**SLLVKYVCCNTDRCN	P60306	32
	LKCNKLVPLFYKTCPAGKNLCYKMFMV**S**NKMVPVKRGCIDVCPK**N**SALVKYVCCNTDRCN	Q98961	33
	LKCNKLVPLFYKTCPAGKNLCYKMFMV**S**NLTVPVKRGCIDVCPK**N**SALVKYVCCNTDRCN	P01442	34
	LKCNKLVPIAYKTCPEGKNLCYKMFMM**S**DLTIPVKRGCIDVCPK**N**SLLVKYVCCNTDRCN	P01451	35
	LKCNKLIPIASKTCPAGKNLCYKMFMM**S**DLTIPVKRGCIDVCPK**N**SLLVKYVCCNTDRCN	P60304	36
	LKCNQLIPIASKTCPAGKNLCYKMFMM**S**DLTIPVKRGCIDVCPK**N**SLLVKYVCCNTDRCN	Q98958	37
	LKCNKLIPIASKTCPAGKNLCYKMFMM**S**DLTIPVKRGCIDVCPK**N**SHLVKYVCCNTDRCN	P79810	38
	LKCNKLIPIASKTCPAGMNLCYKMFMM**S**DLTIPVKRGCIDVCPK**N**SLLVKYVCCNTDRCN	Q91135	39
	LKCNKLPPIASKTCPAGKNLCYKMFMM**S**DLTIPVKRGCIDVCPK**N**SLLVKYVCCNTDRCN	Q98957	40
	LKCNKLIPIASKTCTAGKNLCYKMFMM**S**DLTIPVKRGCIDVCPK**N**SLLVKYVCCNTDRCN	Q91136	41
	LKCNKLIPIAYKTCPEGKNLCYKMMLA**S**KKMVPVKRGCINVCPK**N**SALVKYVCCSTDRCN	P01452	42
	LECNKLVPIAHKTCPEGKNLCYKMFMV**S**TSTVPVKRGCIDVCPK**D**SALVKYVCCNTDRCN	P01454	43
	LECNQLIPIAHKTCPEGKNLCYKMFMV**S**TSTVPVKRGCIDVCPK**N**SALVKYVCCNTDRCN	P01453	44
	RKCNKLVPLFYKTCPAGKNLCYKMFMV**S**NLTVPVKRGCIDVCPK**N**SALVKYVCCNTDRCN	P01443	45
	LKCNKLVPLFYKTCPAGKNLCYKMFMV**S**NKTVPVKRGCIDVCPK**N**SALVKYVCCNTDRCN	O73858	46
	LKCNKLVPLFYKTCPAGKNLCYKMFMM**S**NKTVPVKRGCIDVCPK**N**SALVKYVCCNTDRCN	O73859	47

	DKCNKLVPLFYKTCPAGKNLCYKMFMV**S**DLTVPVKRGCIDVCPK**N**SALVKYVCCNTDRCN	P60311	48
	LKCNKLIPLAYKTCPAGKDLCYKMYMV**S**DKTVPVKRGCIDVCPK**N**SLLVKYECCNTDRCN	P86540	49
	LKCNKLIPLAYKTCPAGKNLCYKMYMV**S**NKTVPVKRGCIDVCPK**N**SPLVKYECCNTDRCN	A0A0U5AL91	50
	LKCNKLIPLAYKTCPAGKNLCYKMYMV**S**NKTVPVKRGCIDVCPK**N**SLLVKYECCNTDRCN	A0A8C6XG05	51
	LQCNKLVPIASKTCPPGKNLCYKMFMV**S**DLTIPVKRGCIDVCPK**N**SLLVKYECCNTDRCN	P86538	52
	LKCNKLIPLAYKTCAAGKNLCYKMYMV**S**NKTVPVKRGCIDVCPK**N**SLLVKYECCNTDRCN	A0A0U4W6K7	53
	LKCNKLIPLAYKTCPAGKNLCYKMYMV**S**NKTVPVKRGCIDVCPK**N**SLLVKYVCCNTDRCN	A0A8C6XQ33	54
	LQCNKLVPIASKTCPPGKNLCYKMFMV**S**DLTIPVKRGCIDVCPK**N**SLLVKYVCCNTDRCN	A0A0U5ARS4	55
	LQCNKLIPLAYKTCPAGKNLCYKMYMV**S**NKTVPVKRGCIDVCPK**N**SLLVKYECCNTDRCN	A0A0U5AR60	56
	LKCNKLVPLAYKTCPAGKNLCYKMYMV**S**NKTVPVKRGCIDVCPK**N**SLLVKYVCCNTDRCN	A0A0U4W6H0	57
	In total: 36 items		
6,	LKCNKLVPLFYKTCPAGKNLCYKMFMV**S**NKMVPVKRGCIDVCPKSSLLVKYVCCNTDRCN	P07525	58
OSO	LKCNKLVPIASKTCPAGKNLCYKMFMM**S**DLTVPVKRGCIDVCPKSSLLVKYVCCNTDICN	Q98956	59
	LECNKLVPIAHKTCPAGKNLCYQMYMV**S**KSTIPVKRGCIDVCPKSSLLVKYVCCNTDRCN	P01448	60
	RKCNKLVPLFYKTCPAGKNLCYKMFMV**S**NLTVPVKRGCIDVCPKSSLLVKYVCCNTDRCN	Q9W6W9	61
	LKCNKLVPLFYKTCPAGKNLCYKMFMV**S**DLTIPVKRGCIDVCPKSSLLVKYVCCNTDRCN	A0A8C6XFH6	62
	LQCNKLVPIASKTCPPGKNLCYKMFMV**S**DLTIPVKRGCIDVCPKSSLLVKYVCCNTDRCN	A0A8C6XFM5	63
	In total: 6 items		
7,	LKCNKLIPLAYKTCPAGKNLCYKMFMVAAPKVPVKRGCIDACPK**N**SLLVKYVCCNTDRCN	P24779	64
OOX	LKCNKLVPLFYKTCPAGKNLCYKMFMVATPKVPVKRGCIDVCPK**N**SLLVKYVCCNTDRCN	Q98959	65
	LKCNKLIPIASKTCPAGKNLCYKMFMVATPKVPVKRGCIDVCPK**N**SLLVKYVCCNTDRCN	Q98962	66
	LKCNKLVPLFYKTCPAGKNLCYKMFMVATPKVPVKRGCIDVCPK**N**SALVKYVCCNTDRCN	Q98960	67
	LKCNKLVPLFYKTCPAGKNLCYKIFMVATPKVPVKRGCIDVCPK**N**SALVKYVCCNTDRCN	Q9DGH9	68
	LKCNKLVPLFYKTCPKGKNLCYKMYMVAAPTVPVKRGCINVCPK**N**SLVLKYECCNTNKCN	newCT[2]	69
	In total: 6 items		
8,	LKCNKLVPLFYKTCPAGKNLCYKMFMVATPKVPVKRGCIDVCPKSSLLVKYVCCNTDRCN	Q02454	70
OOO	LKCKKLVPLFSKTCPPGKNLCYKMFMVATPKVPVKRGCIDVCPKSSLLVKYVCCNTDKCN	P60307	71
	LKCNKLVPLAYKTCPAGKNLCYKMYMVANKKVPVKRGCIDVCPKKSLLVKYECCNTDRCN	P83345	72

	LKCNKLVPLFYKTCPAGKNLCYKMYMVATPKVPVKRGCIDVCPKSSLVLKYVCCNTDRCN	P01440	73
	LKCNKLVPLFYKTCPAGKNLCYKMYMVATPKVPVKRGCIDVCPKSSLLVKYVCCNTDRCN	Q9PST3	74
	LKCNKLVPLFYKTCPAGKNLCYKMYMVAMPKVPVKRGCIDVCPKSSLLVKYVCCNTDRCN	O73856	75
	LKCNKLIPIASKTCPAGKNLCYKMFMVATPKVPVKRGCIDVCPKSSLLVKYVCCNTDRCN	Q91124	76
	LKCNKLVPLFYKTCPAGKNLCYKMFMVAMPKVPVKRGCIDVCPKSSLLVKYVCCNTDRCN	O93473	77
	LKCNKLVPLFYKTCPAGKNLCYKIFMVATPKVPVKRGCIDVCPKSSLLVKYVCCNTDRCN	O93471	78
	LKCKKLVPLFSKTCPAGKNLCYKMFMVAAPHVPVKRGCIDVCPKSSLLVKYVCCNTDRCN	Q9PS34	79
	LKCKKLVPLFSKTCPAGKNLCYKMFMVAAPHVPVKRGCIDVCPKSSLLVKYVCCNTDKCN	P01441	80
	LKCKKLVPLFSKTCPPGKNLCYKMFMVAAPKVPVKRGCINVCPKSSLLVKYVCCNTDKCN	P60309	81
	LKCKKLVPLFSKTCPPGKNLCYKMFMVATPKVPVKRECIDVCPKSSLLVKYVCCNTDKCN	P60308	82
	LKCKKLIPLFSKTCPEGKNLCYKMTMRLAPKVPVKRGCIDVCPKSSFLVKYECCDTDRCN	P25517	83
	LKCNKLVPLFYKTCPAGKNLCYKMFMVATPKVPVKRGCAATCPKSSALVKVVCCKTDKCN	E2ITZ7	84
	LKCHNKLVPFLSKTCPEGKNLCYKMTMLKMPKIPIKRGCTDACPKSSLLVKVVCCNKDKCN	P01471	85
	LKCHNKLVPFLSKTCPEGKNLCYKMTLMKMPKIPIKRGCTDACPKSSLLVKVVCCNKDKCN	B3EWH9	86
	LKCHNKLVPFLSKTCPDGKNLCYKMSMEVTPMIPIKRGCTDTCPKSSLLVKVVCCKTDKCN	P24777	87
	LKCHNKVVPFLSKTCPEGKNLCYKMTLKKVPKIPIKRGCTDACPKSSLLVNVMCCKTDKCN	P24776	88
	LKCNKLVPLFYKTCPAGKDLCYKMYMVATPKVPVKRGCIDVCPKSSLLVKYVCCNTDRCN	P86541	89
	LKCNKLVPLFYKTCPAGKNLCYKMYMVATPKVPVKRGCIDVYPKSSLLVKYVCCNTDRCN	O93472	90
	In total: 21 items		

[1]- Specific residues determining assignment of a CT to the certain group are marked in bold;

[2]- This toxin has not been deposited to any database yet.
